# Are HIV Epidemics among Men Who Have Sex with Men Emerging in the Middle East and North Africa?: A Systematic Review and Data Synthesis

**DOI:** 10.1371/journal.pmed.1000444

**Published:** 2011-08-02

**Authors:** Ghina Mumtaz, Nahla Hilmi, Willi McFarland, Rachel L. Kaplan, Francisca Ayodeji Akala, Iris Semini, Gabriele Riedner, Oussama Tawil, David Wilson, Laith J. Abu-Raddad

**Affiliations:** 1 Infectious Disease Epidemiology Group, Weill Cornell Medical College-Qatar, Cornell University, Qatar Foundation-Education City, Doha, Qatar; 2 Human Development Sector, Middle East and North Africa Region, World Bank, Washington (D. C.), United States of America; 3 HIV Research Section, San Francisco Department of Public Heath, San Francisco, California, United States of America; 4 Department of Social Welfare, School of Public Affairs, University of California, Los Angeles (UCLA), Los Angeles, California, United States of America; 5 Joint United Nations Programme on HIV/AIDS Regional Support Team, Middle East and North Africa, Cairo, Egypt; 6 Regional Office of the Eastern Mediterranean, World Health Organization, Cairo, Egypt; 7 Global HIV/AIDS Program, World Bank, Washington (D. C.), United States of America; 8 Department of Public Health, Weill Cornell Medical College, Cornell University, New York, New York, United States of America; 9 Vaccine and Infectious Disease Institute, Fred Hutchinson Cancer Research Center, Seattle, Washington, United States of America; Johns Hopkins University, United States of America

## Abstract

**Background:**

Men who have sex with men (MSM) bear a disproportionately higher burden of HIV infection than the general population. MSM in the Middle East and North Africa (MENA) are a largely hidden population because of a prevailing stigma towards this type of sexual behavior, thereby limiting the ability to assess infection transmission patterns among them. It is widely perceived that data are virtually nonexistent on MSM and HIV in this region. The objective of this review was to delineate, for the first time, the evidence on the epidemiology of HIV among MSM in MENA.

**Methods and Findings:**

This was a systematic review of all biological, behavioral, and other related data on HIV and MSM in MENA. Sources of data included PubMed (Medline), international organizations' reports and databases, country-level reports and databases including governmental and nongovernmental organization publications, and various other institutional documents. This review showed that onsiderable data are available on MSM and HIV in MENA. While HIV prevalence continues at low levels among different MSM groups, HIV epidemics appear to be emerging in at least few countries, with a prevalence reaching up to 28% among certain MSM groups. By 2008, the contribution of MSM transmission to the total HIV notified cases increased and exceeded 25% in several countries. The high levels of risk behavior (4–14 partners on average in the last six months among different MSM populations) and of biomarkers of risks (such as herpes simplex virus type 2 at 3%–54%), the overall low rate of consistent condom use (generally below 25%), the relative frequency of male sex work (20%–76%), and the substantial overlap with heterosexual risk behavior and injecting drug use suggest potential for further spread.

**Conclusions:**

This systematic review and data synthesis indicate that HIV epidemics appear to be emerging among MSM in at least a few MENA countries and could already be in a concentrated state among several MSM groups. There is an urgent need to expand HIV surveillance and access to HIV testing, prevention, and treatment services in a rapidly narrowing window of opportunity to prevent the worst of HIV transmission among MSM in the Middle East and North Africa.

*Please see later in the article for the Editors' Summary*

## Abstract 2

A systematic review by Laith Abu-Raddad and colleagues collates and analyzes the epidemiology of HIV among men who have sex with men in Middle Eastern and North African countries.

## Editors' Summary

**Background:**

AIDS first emerged in the early 1980s among gay men living in the US. But, as the disease rapidly spread, it became clear that AIDS also affects heterosexual men and women. Now three decades on, more than 30 million people are infected with HIV, the virus that causes AIDS. HIV is most often spread by having unprotected sex with an infected partner and, globally, most sexual transmission of HIV now occurs during heterosexual sex. However, 5%–10% of all new HIV infections still occur in men who have sex with men (MSM, a term that encompasses homosexual, bisexual, and transgender men, and heterosexual men who sometimes have sex with men). In some countries, male-to-male sexual contact remains the most important transmission route. Moreover, although the global prevalence of HIV infection (the proportion the world's population infected with HIV) has stabilized, the prevalence of HIV infection among MSM seems to be increasing in multiple countries and new and resurgent HIV epidemics among MSM populations are being frequently reported.

**Why Was This Study Done?:**

In the US and the UK, the MSM population is visible and has helped to raise awareness about the risks of HIV transmission through male-to-male sexual contact. In many other countries, MSM are much less visible, fearing discrimination, stigmatization (being considered socially unacceptable) or arrest. In the Middle East and North Africa (MENA, a geographical region that encompasses countries that share historical, socio-cultural, linguistic and religious characteristics), MSM are the most hidden HIV risk group. Consequently, very little is known about HIV transmission patterns among MSM in MENA. Indeed, it is widely thought that there is virtually no information available on the epidemiology (causes, distribution, and control) of HIV among MSM in this region. In this systematic review and data synthesis, the researchers use predefined search criteria to identify all the published and unpublished data on the epidemiology of HIV among MSM in MENA and combine (synthesize) these data to produce a coherent picture of the HIV epidemic in this potentially key group of people for HIV transmission in this region.

**What Did the Researchers Do and Find?:**

The researchers identified 26 articles and 51 other country-level reports and sources of data that included data on the prevalence of male-to-male sexual contact, HIV transmission, levels of high-risk behavior, and the extent of knowledge about HIV among MSM in MENA. The prevalence of HIV infection among MSM was low in most countries but high in others. For example, the infection rate in Pakistan was 27.6% among one MSM group. Importantly, there was some evidence of increasing HIV prevalence and emerging epidemics among MSM in the region. Thus, by 2008, MSM transmission was responsible for more than a quarter of notified cases of HIV in several countries. Worryingly, MSM were involved in several types of HIV-related high risk behavior. For example, they had, on average, between 4 and 14 sexual partners in the past six months, their rates of consistent condom use were generally below 25% and, in some countries, MSM frequently reported injecting drug use, another common mode of HIV transmission. In addition, 20%–75.5% of MSM exchanged sex for money and contact between MSM and female sex workers and other female sexual partners was often common. Finally, although the level of basic knowledge about HIV/AIDS was high, the level of comprehensive knowledge was limited with a high proportion of MSM perceiving their risk of contracting HIV as low.

**What Do These Findings Mean?:**

These findings indicate that there is considerable and increasing data about HIV transmission and risk behavior among MSM in MENA. However, the quality of this evidence varies greatly. Little has been collected over time in individual populations and, because only the visible part of the MSM populations in many MENA countries has been sampled, these findings may not be representative of all MSM in this region. Nevertheless, these findings suggest that HIV epidemics are emerging among MSM in several MENA countries. Importantly, the high levels of risk behaviors practiced by many MSM in MENA mean that MSM could become the pivotal risk group for HIV transmission in this region in the next decade. There is, therefore, an urgent need to expand HIV surveillance and access to HIV testing, prevention and treatment services among MSM in this region to limit the size of the HIV epidemic.

**Additional Information:**

Please access these Web sites via the online version of this summary at http://dx.doi.org/10.1371/journal.pmed.1000444.

- Information about the status of the HIV epidemic in the Middle East and North Africa can be found in the World Bank/UNAIDS/WHO report Characterizing the HIV/AIDS epidemic in the Middle East and North Africa: Time for strategic action

- Information about the global HIV epidemic among men who have sex with men can be found in the World Bank report The Global HIV Epidemics among Men Who Have Sex with Men
- Information is available from the US National Institute of Allergy and Infectious Diseases on HIV infection and AIDS
- HIV InSite has comprehensive information on all aspects of HIV/AIDS, including information on HIV transmission and transmission in gay men and other MSM and on safer sex
- Information is available from Avert, an international AIDS charity, on all aspects of HIV/AIDS, including information on HIV, AIDS and men who have sex with men and on HIV and AIDS prevention (in English and Spanish)
- The US Centers for Disease Control and Prevention also have information about HIV/AIDS among men who have sex with men (in English and Spanish)

## Introduction

Male-to-male sexual contact is one of the leading modes of HIV transmission [1]–[3]. While the global prevalence of HIV has stabilized [1], there seems to be a trend of increasing HIV prevalence among men who have sex with men (MSM), and recent reports indicate new, newly identified, and resurging HIV epidemics among MSM in different parts of the world [2]–[3]. In North America, MSM transmission continues as the leading contributor to the HIV epidemic, with studies indicating resurgent epidemics among at least some MSM populations [4]. In sub-Saharan Africa, recent studies are unveiling hidden HIV epidemics among MSM [1]–[2],[5]–[7]. Emerging and growing HIV epidemics among MSM have been recently identified in several countries in East, Southeast, and South Asia, as well as in Latin America [1]–[2],[8]. In several countries in East Asia, male-to-male sex has become the dominant mode of transmission among newly diagnosed infections [2].

MSM form the most hidden and stigmatized of all HIV risk groups in the Middle East and North Africa (MENA). It is reported that they are often subject to homophobia, harassment, discrimination, and criminalization [9]–[13]. Homosexual characters have been documented in different epochs of Middle Eastern history and are well described, including prominent political and literary historical figures [14]–[16]. Arab literature, both classical and modern, has portrayed MSM characters in varying lights and contexts [17]–[18]. For much of history, and despite sociocultural prohibitions against homosexuality [12], Arab and Islamic cultures tended to treat homosexuals somewhat with indifference, and there does not appear to be historically an ideology of homophobia similar to that that existed in other regions [19]–[20]. In Medieval times, the apparent indifference to homosexuality in this region was viewed in the West as a sign of moral decadence [19]. The emergence of an ideology of homophobia in MENA cannot be understood without elaborating the historical context of colonialism, rise of the modern nation state, establishment of penal codes modeled over European jurisdiction, the politics of exclusion by the state, and more recent reactions to external influences in the modernization of societies [19],[21]–[23].

MENA remains the only region where knowledge of the HIV epidemic continues to be limited and subject to much controversy [24]. The region continues to be viewed as “a real hole in terms of HIV/AIDS epidemiological data” [25], particularly so with respect to MSM data which are characterized as being “virtually absent” [26]. The sociocultural and political challenges engulfing MSM in this region may limit access of this population to HIV prevention services thereby suggesting the potential vulnerability of this population to HIV. This systematic review delineates the evidence on the epidemiology of HIV among MSM in MENA, including the prevalence of male-to-male sex, HIV transmission among MSM, levels of risk behavior, and extent of HIV knowledge among MSM in MENA. It provides an integrated analysis and synthesis of the evidence to address the gap in our knowledge of what potentially could materialize as the key risk group for HIV sexual transmission in this region in the next decade.

## Methods

### Data Sources and Search Strategy

The PRISMA checklist can be found in Text S1. This work was part of a comprehensive systematic review of all available data about HIV in MENA, which was complemented by another detailed search focused specifically on published MSM literature in MENA. The search strategies under each one of these two approaches are summarized below, with more details on the search criteria in Text S2:

#### (A) Comprehensive review of HIV data in MENA

The main source of data for this investigation was the MENA HIV/AIDS Epidemiology Synthesis Project [27], the mandate of which was to collect and synthesize all available data on HIV, sexually transmitted infections (STIs), and sexual risk behavior in MENA. Data on all high-risk groups were retrieved, including MSM, injecting drugs users (IDUs), and female sex workers (FSWs), in addition to data on bridging and general populations. The project was conducted through a partnership of the World Bank, the MENA Regional Support Team of the Joint United Nations Programme on HIV/AIDS (UNAIDS), and the Eastern Mediterranean Regional Office (EMRO) of the World Health Organization (WHO). The project was concerned with MENA as a geographical region encompassing countries that share historical, sociocultural, linguistic, and religious characteristics, and thus covered all 23 countries included in the MENA definitions of these three partner organizations. The list of included countries can be found in Text S3.

The data sources identified as part of our comprehensive search of relevant studies and databases included a scientific literature search of PubMed (Medline), peer-reviewed publications in local and regional research journals, international organizations' reports and databases, country-level reports and databases, governmental and non-governmental organizations' studies and publications, and other institutional reports related to HIV and STIs in MENA. More details on these search criteria can be found in Text S2.

#### (B) Specific data about MSM in MENA

The above generic search was complemented by a specific search of all data on MSM in MENA in the biomedical literature. The data sources that were searched included the PubMed database using “homosexuality” and “bisexuality” as MeSH terms and keywords, the Embase database using the *Emtree* terms “homosexuality” and “bisexuality”, and regional online databases, namely the WHO African Index Medicus (AIM) [28], the WHO Index Medicus for the Eastern Mediterranean Region (IMEMR) [29], and the Scientific and Technical Egyptian Bibliographic Database (STEB) [30]. Details of the search criteria for each of these databases are in Text S2.

### Study Selection

The titles and abstracts of all records retrieved were screened for relevance independently by two of the authors (GM and LA-R). Bibliographies of relevant articles were cross-checked for other potential citations. In this first review of HIV and MSM from a region perceived to have very limited data on this topic [25]–[26], inclusion criteria were kept broad to cover all relevant data from the region. The data were categorized according to quality of study design and methods at a later stage in the analysis phase as described below. At the initial screening phase, eligibility was based on having a data point on any of the following measures among MSM in any of the 23 MENA countries covered in our search: HIV infection prevalence or incidence, other STI prevalence, hepatitis C virus (HCV) infection prevalence, the prevalence of any sexual or injecting risk behavior measure, and data on knowledge and attitudes towards HIV/AIDS. Studies were also eligible for inclusion if they reported data on the prevalence of male same-sex sexual behavior, MSM population size estimates, and the contribution of anal sex as a mode of HIV transmission among notified and diagnosed HIV/AIDS cases.

### Data Extraction

Data on the above indicators were extracted from relevant records independently by two of the authors (GM and LA-R). Disagreements were settled by discussions within the study team and in some instances by contacting the authors or country-level program managers/sponsors of the study for further clarification. Articles in Arabic were reviewed by the same two authors, whose first language is Arabic. Articles in French (mostly from Francophone North African MENA countries) were screened and handled for data extraction by one of the authors fluent in French (GM) and by a French-speaking epidemiology consultant. There were few relevant records in other languages, and data in these articles were abstracted from the English abstract.

### MSM Definition

To conform with terminology standards commonly used in HIV literature [31], the term “men who have sex with men” was used in this article to refer to the population of men who engage in same-sex sexual activities, specifically anal sex, regardless of their gender, sexual, social, or cultural identity. This population includes men who describe themselves as homosexuals, bisexuals, male sex workers, transgender, or any other country-specific population of MSM. Since very few studies in the available literature in MENA differentiate between MSM and transgender subgroups, and since, as will be described later, sexual and gender identities in this region are complex and often not distinctly defined, the emphasis was on being as inclusive as possible of all studies of male-to-male sexual behavior in the region, which often combined persons of different sexual orientations and gender identities, levels of same-sex attraction, and levels of sexual behavior with men and women.

Our inclusion criteria were thus based on the mode of disease transmission rather than the ethnographic and social background of the term. Nuances between the different types of MSM populations were made in the text as appropriate and warranted by the data. Particular attention has been made to distinguish, when possible, transgender people from the rest of the MSM population groups. It is worth noting that transgender people are more visible in specific parts of MENA, mainly Pakistan [32], compared to the rest of the countries. Therefore studies in Pakistan usually distinguished this population group from the rest of MSM groups as opposed to the rest of MENA countries where the tendency has been to include the transgender population within the wider MSM definition.

## Results

**Figure 1:**
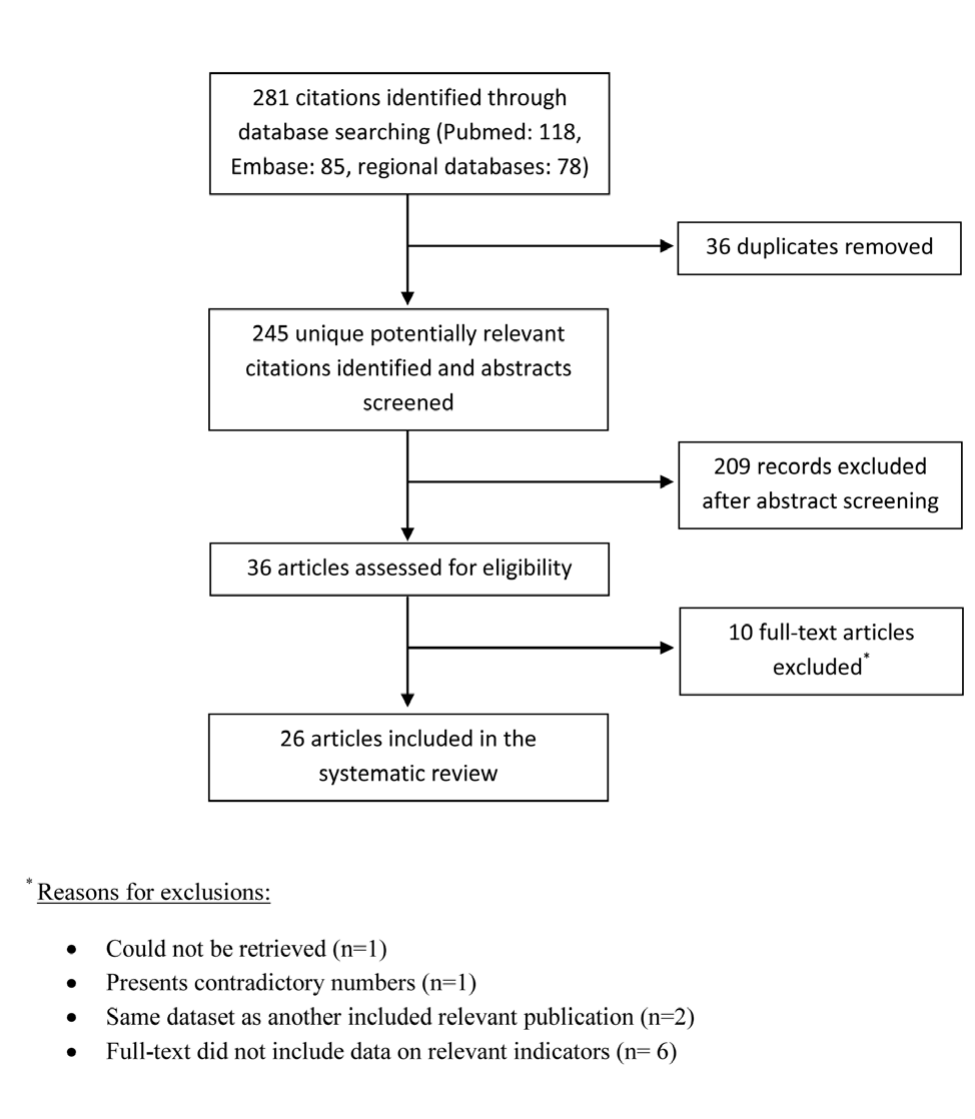
Flow of article selection for the specific MSM in MENA search of scientific databases. This chart, adapted from PRISMA 2009 flow diagram, displays the flow of article selection for the specific MSM in MENA search of scientific databases, namely PubMed, Embase, and regional databases. Further relevant studies included in the review, mainly in the form of country-level reports, were identified through the comprehensive search of the MENA HIV/AIDS Epidemiology Synthesis Project.

The study selection process, described in Figure 1, was adapted from PRISMA 2009 flow diagram [33]. Out of a total of 118 records retrieved through PubMed and 85 records retrieved through Embase for the specific MSM in MENA searches, 23 publications were selected as relevant for the present article. Only three relevant documents were not covered by the PubMed and Embase searches and were identified out of the 78 records retrieved through the search of the three regional databases (AIM, IMEMR, and STEB). The remaining relevant documents were identified through the extensive and multi-source data search of the Synthesis Project. These include: 15 publications identified through the HIV in MENA generic search of PubMed, three international organization reports, 43 country-level reports, in addition to eight other miscellaneous documents that were included in the present article.

### Context of Male Same-Sex Sexual Behavior in MENA

In MENA, sexual identities are complex and fluid, and homosexuality takes multiple forms beyond the usually recognized rigid distinction between homosexuals and heterosexuals in contemporary Western culture. As opposed to the “gay” system [34], a large fraction of same-sex sexual practices among males in MENA takes place between males who do not identify themselves as having a same-sex sexual orientation, and who usually do not consider themselves as gay, homosexuals, or bisexuals [22]–[23],[35]–[36]. The boundaries of sexuality are generally not sharply defined and same-sex sexual activity at times may occur as an occasional alternative to male–female sexual activity [35]. The perception that only males who engage in receptive anal sex are considered homosexuals is also common among MENA populations [13],[34],[36]. Overall, Arab/Islamic societies seem to put more emphasis on the sexual act itself rather than the sexual orientation or identity of those engaged in the sexual act [35], and there is an ongoing debate about the nature and functionality of MSM identities within the sociocultural context of MENA [22]–[23].

An example of the complexity of male same-sex behavior and gender identities is portrayed in Pakistan, where there is a complex tapestry of MSM activity and male sex work [19]. These include *hijras* or *khusras* (transgender people who dress as women), *khotkis* (biological males who dress publicly as men but have “female souls” and feminized traits), *banthas* (biological males with a male gender identity), and *giryas* (husbands of *hijras*; have a male gender identity), among other forms [37]–[39]. Another example is in Yemen, where distinct typologies of MSM identities exist such as the “*khwaniya*” (biological males with feminized traits) and “*khafya*” (bisexual males who hide their same-sex sexual orientation) [36].

Same-sex sexual activity and orientation is viewed negatively among MENA populations, and studies have documented negative attitudes and perceptions towards homosexuality [40]–[43]. There are strong religious prohibitions in Islam against male same-sex practices, but the different schools of Islamic jurisdiction differ on the context of prohibition and its consequences [35],[44]. Male same-sex practices are also generally illegal in MENA, and the penalties range from capital punishment in at least five MENA countries to no explicit stipulated punitive measures in two countries [35]. Most countries, however, include prison time in their penal codes that ranges from one to 10 years in prison [35]. Despite some notable exceptions [9]–[12],[45], it is rare for MENA states to enforce the penal code and persecute MSM strictly for their sexual practices [35].

### Prevalence of Male Same-Sex Sexual Behavior in MENA

Estimating the prevalence of male same-sex sexual behavior is difficult in most countries and cultural contexts [46], particularly in MENA where stigmas associated with homosexuality and HIV, in addition to poor health research capacity (among other factors [47]), have led to a scarcity of reliable estimates. However, the MSM populations in MENA are becoming progressively more visible and there has been a considerable increase in the knowledge of MSM in this region [27].

The proportion of the male population engaging in anal sex with males in MENA (Table 1) seems to be consistent with reported global levels of very roughly 2%–3% [26],[46],[48]–[51]. For example, same-sex sexual contacts were reported by 3.7% [52] and 2.0% [51] of male youth in Morocco and Sudan, respectively. Population size estimates for MSM were available from five governorates in Yemen indicating a proportion ranging between 0.39% and 1.62%, with a national estimate of 44,320 MSM [53]. Higher rates of anal sex activities have been reported in several select populations in MENA including truck drivers, street children, and prisoners (Table 1). Previous reports from countries in different parts of the world have also reported similar higher male-to-male sex rates among certain male populations such as truck drivers [26],[54].

**Table 1:** Prevalence of anal sex between males in select populations in MENA.

Population	Egypt	Lebanon	Iran	Morocco	Pakistan	Sudan	Yemen
Male youth				3.7% [52]		2.0% [51]	
Males in VCT centers	3.5% [97]						
Unmarried sexually active men			29%^a^ [125]				
Population size estimate							0.61%–1.47% [53]
Truck drivers				9% [126]	11.3%^b^ [127], 21.9% [61], 49.3% [127]	0.5% [128]	
Truck drivers at STD clinics					53% [129]		
Male street children	15.0%^c^ [71], 28.3% [130], 37.1%^c^ [70], 44.2% [71], 77.4% [70]						
Prisoners		8.4% [121]			26.0%^d^ [131]	2.2% [132]	
Migrants					1.7% [133]		

This table summarizes available data on the prevalence of anal sex between males in select populations at varying levels of risk of exposure to HIV.

a Unspecified male-to-male sexual contacts.

b With a MSW.

c Forced anal sex.

d Before incarceration.

VCT, voluntary counselling and testing.doi:10.1371/journal.pmed.1000444.t001

Gender segregation, delayed marriage, difficulty in accessing females for sex, and overcrowded living conditions have been suggested as contributors to casual, male same-sex, anal sexual contacts [12],[55]. The existence of separate social spheres for men and women in MENA societies, and the sharp boundary in the Arab and Islamic culture between the private and public spheres, have been suggested as sociocultural attributes that may facilitate same-sex sexual practices without drawing suspicious attention from the public eye [35]. The home in MENA societies is seen as an independent and self-governed entity and is traditionally beyond the realm of the state [56]. Yet, we found little evidence to support or refute any distinguishing specificity of MENA societies in relation to the prevalence of male same-sex practices. The prevalence of these practices appears, within the limitations of available data, to be consistent with that in other regions.

### HIV Prevalence among MSM in MENA

HIV transmission among MSM has been documented in most MENA countries. Sources of data include various studies and surveys (Table S1) as well as countries' case notification reports (Table 2). Data from available studies indicate that the practice of anal sex contributes by considerable proportions to HIV transmission of up to 26% in a number of MENA countries such as in Egypt [57]–[58], Lebanon [59], and Yemen [60] (Table S1). Also, countries' case notification reports reveal that homosexual/bisexual sex contributed cumulatively about 13% of HIV infections in Egypt, Lebanon, and Oman since the first HIV/AIDS diagnoses in these countries, whereas the cumulative MSM contribution in other countries seems less prominent (Table 2). Still, in most countries with available data, case notification reports indicate a trend of increasing proportions of HIV cases due to MSM transmission in recent years as compared to cumulative data since the first HIV/AIDS diagnosis in each country (Table 2). For example, in Lebanon, MSM mode of transmission accounted for 52.3% of notified HIV cases in 2008, at a time when the cumulative proportion of infections due to MSM transmission was only 13.0%. Pakistan is the only country where apparently the reverse trend was observed, but this is due to the recent rapid increase in the denominator of HIV infections among IDUs in this country [61]–[66].

**Table 2:** Contribution of MSM mode of transmission to the total diagnosed and notified HIV/AIDS cases by country as per countries' case notification reports.

Country	Report Period	Countries' Case Notification Reports					
		Most Recent Available Report			Cumulative		
		n	N	*%*	n	N	*%*
Bahrain	All 2008	1	21	*4*.*8*	9	183	*4*.*9*
Egypt	1^st^ quarterly report 2008	12	61	*19*.*7*	297	2,242	*13*.*2*
Iran	4^th^ quarterly report 2007	0	29	*0*.*0*	0	16,679	*0*.*0*
Iraq	All 2008	0	5	*0*.*0*	0	274	*0*.*0*
Lebanon	All 2008	11	21	52.3	63	483	13.0
Oman	2^nd^ quarterly report 2007	5	20	*25*.*0*	206	1,585	*13*.*0*
Pakistan	3^rd^ quarterly report 2008	3	216	*1*.*4*	123	4,755	*2*.*6*
Saudi Arabia	4^th^ quarterly report 2008	0	109	*0*.*0*	26	2,291	*1*.*1*
Syria	4^th^ quarterly report 2008	2	7	*28*.*6*	6	339	*1*.*8*
Tunisia	4^th^ quarterly report 2008	1	8	*12*.*5*	73	1,499	*4*.*9*

When more than one country report was available, only the most recent one was used.

A comparison of the percentage of notified infections that is due to MSM transmission between the most recent available report and cumulatively up to that report hints at recent trends (whether increasing, decreasing, or similar) in the contribution of MSM mode of transmission in each country.

Notes: MSM Includes homosexuals and bisexuals. Cumulative: Since the beginning of the epidemic and until the most recent available report. n, number of positive cases among MSM; N, Total number of positive cases; %, percentage of MSM HIV-positive cases out of the total number of HIV positive cases.

HIV point-prevalence measures among MSM in MENA, including male sex workers (MSWs) and transgender people, have been documented in various sources of literature including scientific peer-reviewed publications, country level reports and databases, and international organization databases. These data were reviewed and organized according to the quality of the evidence. Studies using well-defined or state-of-the art surveillance methodologies are described in detail in Table 3, whereas point-prevalence surveys with no sufficient information on the methodologies adopted or the populations studied are summarized in Table S2.

**Table 3:** HIV prevalence among MSM in MENA as reported by studies with well-defined methodologies.

Country	Study	Year	Sampling	Study Site	Population	Sample Size	HIV Prevalence
**Egypt**	El-Sayyed N, 2008 [76]	2003	SBS	Cairo	MSM	73	1.4%
	Ministry of Health, 2006 [70]	2006	RDS	Alexandria	MSM	267	6.2%
	Ministry of Health, 2010 [71]	2010	RDS	Cairo	MSM	259	5.7%
				Alexandria	MSM	262	5.9%
				Luxor	MSM	268	0%
**Iran**	Eftekhar M, 2008 [67]	2007	RDS	Tehran	Homeless MSM	101	14.8%
**Jordan**	National AIDS Program, 2010, [80]	2008	RDS	Four cities	MSM	468	0.2%^a^
**Lebanon**	Mishwar, 2008 [83]	2007-8	RDS	Beirut	MSM	101	3.7%
**Morocco**	National AIDS Program, 2008 [134]	2008	CvS	Agadir and Marrakech	MSM	90	4.4%
**Pakistan**	Khanani, 2010 [68]		RDS	Karachi	MSM	396	11.4%
	Bokhari, 2007 [61]	2004	MSWs: RDS	Karachi	MSWs	409	3.9%
	Khan, 2008 [78]		*Hijras* c: CS		*Hijras* c	199	1.5%
				Lahore	MSWs	400	0.0%
					*Hijras* c	204	0.5%
	Altaf, 2006 [135]	2005	RDS	Karachi	MSWs	199	7.0%
					*Hijras* c	199	2.0%
	National AIDS Program, 2005 [64]	2005	MSWs: RDS	Eight sites	MSWs	1,779	0.4% (0.0–4.0%)^b^
			HSWs^c^: CS		HSWs	1,469	0.8% (0.0–1.6%)^b^
	National AIDS Program, 2006-7 [63]	2006	MSWs: RDS	12 sites	MSWs	2,289	1.5% (0.0–7.5%)^b^
			HSWs^c^: CS		HSWs	2,143	1.8% (1.0–14.0%)^b^
	Hawkes, 2009 [39]	2007	RDS	Rawalpindi	*Khusra* c	253	2.4%
					*Khotkis* c	364	0.0%
					*Banthas* c	195	0.5%
				Abbottabad	*Khusra* c	16	0.0%
					*Khotkis* c	4	0.0%
					*Banthas* c	83	0.0%
	National AIDS Program, 2008 [62]	2008	MSWs: RDS	Six sites	MSWs	1,200	0.9% (0.0–3.1%)^b^
			HSWs: CS		HSWs	1,181	6.4% (0.0–27.6%)^b^
**Sudan**	Elrashied, 2006 [69],[84]	2005	SBS	Khartoum	Receptive MSM	713	9.3%
	Elrashied, 2008 [79]	2007	SBS	Khartoum	Insertive MSM	406	7.8%
**Tunisia**	National AIDS Program, 2010 [13]	2009	RDS	Three regions	MSM	1,778	4.9% (0.8–6.3%)^b^

These studies present the best available evidence on the prevalence of HIV among MSM as most of them are integrated bio-behavioral surveillance surveys and use state of the art sampling methodologies for hidden and hard to reach populations. A description of the different sampling methodologies displayed in the table can be found in Table S6. All studies were cross-sectional by design.

a Sample proportion.

b Average of different cities (range).

c *Banthas*: biological males with a male gender identity; *Giryas*: husbands of *hijras*; *Hijras* and *khusras*: transgender people; *Khotkis*: biological males who dress as men but have “female souls” and feminized traits.

CS, one-stage cluster sampling; CvS, convenient sampling; RDS, respondent-driven sampling; SBS: snow-ball sampling.

The available point-prevalence levels from recent and methodologically sound studies (Table 3) indicate a considerable HIV spread among certain MSM groups in MENA with rates reaching up to 14.8% in Iran [67], 27.6% in Pakistan [68], and 9.3% in Sudan [69]. In Egypt, the two rounds of surveillance in 2006 and 2010 consistently indicate concentrated levels of HIV at a prevalence of about 6% [70]–[71]. A substantial intracountry variability in HIV prevalence among different MSM groups is apparent, such as in Egypt [71], Pakistan [62]–[64], and Tunisia [13]. There also seems to be a trend of increasing HIV prevalence among MSM in more recent studies compared to earlier investigations. This can be seen, for example, among *hijra* sex workers (HSWs) in Pakistan, where a steady increase in HIV prevalence has been observed in the three rounds of the multicenter integrated biobehavioral surveillance surveys from 0.8% in 2005 to 1.8% in 2006 to 6.4% in 2008 (Table 3) [62]–[64]. On the other hand, most countries report no or very limited HIV prevalence among MSM for the earlier years with available data (Table S2). This pattern of no or very limited HIV prevalence appears to persist in several MENA countries (Table S2 and Table 3).

### Risk Behavior among MSM in MENA

Evidence indicates that MSM in MENA are involved in HIV-related risk behaviors including multiple and concurrent sexual partnerships, low or inconsistent condom use, overlap with injecting drug use risk behaviors, and involvement in heterosexual sex with FSWs and/or other females through spousal and nonspousal partnerships.

#### Sexual behavior and partnerships

Table S3 summarizes available sexual risk behavior measures among MSM in MENA countries. In most MSM populations surveyed, over 90% reported having multiple partners [72]–[74], some of which were concurrent in time, with new partnerships being formed while existing partnerships still have not dissolved, or concurrent in the same coital episode (group sex) [73],[75]–[76]. The number of partners per MSM varied between sites and ranged from an average of four to 14 partners in the last six months (Table S3), with numbers reaching up to 148 partners among some MSM [74].

MSM in MENA reported different forms of sexual partnerships, including steady, casual, and commercial. Among available studies, the majority of MSM report having steady partners and anal sex with casual non-commercial partners (Table S3). Paying for anal sex with commercial sex partners was also reported with rates ranging between 12.0% and 80.3% [74],[77]–[79] (Table S3). Oral sex was commonly practiced, with most of the acts being unprotected [13],[69],[78]–[80]. The proportions of MSM engaged in receptive and insertive anal sex were 49%–90.6% and 74%–95.4%, respectively (Table S3). MSM in MENA also engage in noncommercial partnerships with MSWs, which exposes them to higher risk of HIV infection, as seen in Pakistan among MSWs and HSWs [62]–[64] (Table S3).

#### Condom use

Reported condom use among MSM in MENA varies substantially though overall it is on the low side especially in resource limited settings (Table S4). With the exception of Lebanon and Oman [77],[81]–[82], rates of consistent condom use among MSM in MENA were generally below 25% among surveyed MSM in multiple studies [39],[62]–[64],[72],[76] (Table S4). The lowest rates of condom use at last sex were reported in Egypt and in Pakistan, while the highest rates were reported in Lebanon, Tunisia, and Sudan, reaching up to 70% (Table S4) [69],[74],[83]. Rate of lubricant use during anal sex differed between countries: 38.7% in Jordan [80], 18.3% [74] to 73.5% [13] in Tunisia, about 50% in Pakistan [63], and 88.8% in Sudan [69].

While general knowledge of condoms in MENA was high, with over 80% of MSM having heard of condoms [13],[74],[76],[78], knowledge of their protective effects was much lower, in the range of 31.1%–50.7% [62]–[64],[69],[76],[78],[80], with the exception of Lebanon where 96% of MSM knew that using condoms during anal sex prevents HIV transmission [83]. Overall, unavailability and disliking condoms were the main reasons cited for not using condoms in MENA. Twenty-two percent of MSM in Egypt [72], and as much as 70.5% in Tunisia [74] and 85.3% in Sudan [84], reported difficulty in obtaining condoms [74]. Disliking condoms, with the perception that they reduce the sense of pleasure, was reported as a reason for nonuse by 13.7%–62% of MSM in multiple studies [62],[67],[74],[76]. Overall, however, the frequency of condom use among MSM in MENA is comparable and within the range reported in other regions [26].

#### Overlap with other high-risk groups

MSM risk behaviors in MENA overlap considerably with other high-risk groups such as IDUs and FSWs. Overlap of high-risk groups is a key factor in emerging HIV epidemic dynamics, as it allows an infection already established in one risk group to be bridged to another high-risk group. This process is best portrayed in Iran, where injecting drug use is the main mode of HIV transmission, but recently an HIV prevalence of 14.8% has been reported among street-based MSM in Tehran [67]. As many as 53.0% of these MSM reported injecting drugs in the last month [67]. The elevated HCV prevalence among this MSM group at 48.8% further affirms the overlap between MSM and injecting drug risk factors [67], and suggests that the overlap may have fueled HIV transmission among MSM in Iran. It is worth noting that phylogenetic analyses of IDU isolates in Iran showed confined variability in HIV1 genes, arguing for the recency of the IDU epidemic in Iran [85]–[87], and further affirming that the observed prevalence among MSM is rather recent. Recent phylogenetic evidence from Pakistan has also linked the emerging epidemic among MSM to that which emerged among IDUs earlier in this decade in this country [88].

MSM in other MENA countries also report considerable levels of injecting drug use. These are summarized in Table S5, which displays the measures of overlapping MSM and IDU risk behaviors in MENA. It is worth noting that it is not clear whether these levels of injecting drug use among MSM reflect individuals who identify primarily as being MSM and practice injecting drug use, or whether these are primarily IDUs who have been sampled in the epidemiological studies as MSM because they are engaged in anal sex for drugs or money to sustain their injecting practices. Indeed, and as can be seen in Table S5, a considerable proportion of male IDUs in MENA are also involved in same-sex practices, probably for the exchange of sex for drugs or money, with rates reaching more than 30% in some settings [82],[89]–[91]. The overlap between MSM and IDUs in MENA occurs among both injecting and sexual networks, but appears to be more focused on sexual networking rather than on drug networking [92]. It is worth noting that the subgroup of MSM IDUs appears to be at high risk of HIV infection, not only due to exposure to two modes of HIV acquisition (sexual and injecting), but also because this population appears to engage in higher levels of sexual and injecting risk behaviors compared to the populations who engage in only one type of risk behavior [93].

Overlap of MSM activities with heterosexual commercial sex networks is also common in MENA where contact with FSWs was reported at various levels among MSM. For example, in Egypt and Tunisia, 38.4% [76] and 48.2% [13] of MSM, respectively, reported ever having had sex with a FSW. In Pakistan, several studies documented that 12.3%–42.7% of MSWs paid a female for sex in the last month [61]–[62].

#### Male sex work

Sex work appears to be common among studied MSM in MENA. Overall, exchanging sex for money was reported by 20%–75.5% of MSM [69]–[70],[75],[83]–[84], with higher rates reaching almost 100% being reported among transgender people (*hijras*) in Pakistan [61],[78] (Table S3). These proportions are comparable, though on the high end, to global ranges of 5%–76% of MSM engaging in sex work [26]. This in part reflects that MSWs might be the most visible MSM subpopulation in MENA and that surveillance work may have not yet penetrated deeply into the larger MSM sexual networks [36].

MSWs and HSWs population size estimates were available for Pakistan. In 2005, there were an estimated average of 2.3 MSWs and 2.4 HSWs per 1,000 adult men across 12 cities [64]. The range of prevalence of sex work varied from 1 to 4.8 per 1,000 adult men for MSWs and 0.4 to 3.7 per 1,000 adult men for HSWs in these cities [64]. Data from a more recent mapping indicate that there were 0.6 to 7.4 MSW and 0.1 to 4.9 HSW per 1,000 adult men across 13 cities in Pakistan in 2006, with national estimates of 1.7 MSWs and 0.9 HSWs per 1,000 adult males [94].

The nature of commercial sex work among MSM in MENA appears to be changing. Increasingly MSWs and HSWs in Pakistan find their clients by seeking them in public places like bus stops and markets, using mobile phones, or through client referral instead of more “traditional” modes through a pimp or a *guru* (the patron of a small group of *hijras*) [62]–[64]. Similarly in Yemen, MSWs often practice sex work in parks, cafés, market places, and near sea ports, in addition to hotels, bars, and private houses [36]. There is also some evidence that male and female commercial sex networks are interlinked. A study among FSWs in Sudan reported that FSWs host young MSWs in their houses to offer both opposite-sex and same-sex sex to clients who wish to engage in both activities [95]. Also in Yemen, private houses offering MSW services are frequently run by women, known as “*kawada*”, who often are female sex workers themselves [36].

MSWs in MENA report relatively high levels of sex work activity with an average of 1.9–3 clients per working day and 20.3–49.1 clients per month, as reported in studies from Pakistan [62]–[64] and Afghanistan [96]. In one study in Sudan, 55.6% of receptive MSWs reported having 20–40 clients in the last six months [69]. The average number of clients was higher among HSWs than MSWs in Pakistan [62]–[64]. The mean number of years in sex work was reported to be between 6.7 and 12.5 years in this country [39].

### Engagement of MSM in Heterosexual Sex with Noncommercial Female Partners

Some MSM in MENA appear to engage in opposite-sex in addition to same-sex practices. Given the apparently rising HIV epidemics among MSM in MENA, this might put female sexual partners of MSM at elevated levels of HIV exposure. Several studies have reported that nearly 5%–52% of MSM and MSWs in MENA have ever been married [13], and 3%–35% are currently married [9],[13],[39],[62]–[64],[69]–[71],[74]–[75],[78],[80],[83], although this may not necessarily imply an active sexual life with the female spouse.

A substantial proportion also report having nonspousal as well as spousal sexual relations with females. In Egypt, Lebanon, Tunisia, and Sudan, 39.8%–86.5% [71], 52.5% [77], 69.4% [13], and up to 69% [69],[73] of MSM reported ever having sex with a female, respectively. Also in Egypt, 33% [97] and 44% [75] of MSM were found to be bisexually active. In Iran and Jordan, 87.7% [27] and 65.3% [80] of MSM reported having female partners in the last six months, respectively, while 9%–24% of MSWs in Pakistan had sex with a nonpaying female in the last month [61]. In Tunisia, 31.1% of MSM reported that their last sex act was with a female [74], and 25.0% of MSM reported more than five female sexual partners in the last six months [13]. These proportions of marriage and opposite-sex sexual contacts among MSM in MENA are consistent with the ranges reported in other regions [26].

### Knowledge of HIV/AIDS

Levels of HIV/AIDS knowledge among MSM appear to vary in MENA. Overall, the rate of basic knowledge about HIV was high in the region, with the proportion of MSM reporting having ever heard of HIV being 82.2%–99.5% in Egypt [71],[76] (with the exception of one setting reporting 33.3% [71]), 82.4% in Iran [27], 85.1% in Jordan [80], 66.1%–85.2% in Pakistan [62]–[64], 96.1% in Tunisia [13], and 96.4% in Sudan [69].

With the exception of Lebanon and Tunisia, where the majority of MSM were aware of HIV, its modes of transmission, condom role in prevention, and other prevention measures [13],[77],[81],[83], levels of comprehensive HIV knowledge among MSM in the region were overall limited [69],[98], and several misconceptions were noted among various MSM groups [69]–[70],[74],[80]. For example, some MSM in Sudan reported the belief that limiting activity to an insertive role prevents fully HIV infection, and most MSM believed-as has been found in other settings in Africa and Asia [26]—that the risk of contracting HIV through anal sex is far less than that in vaginal sex [69].

It is of particular concern that the proportion of MSM in MENA who perceive themselves at risk of contracting HIV was low despite relatively good knowledge in some settings. For example, 43.3% [77],[81] and 33% [83] of MSM in Lebanon and 46.6% in Egypt [76] perceived no chance of acquiring HIV. In Pakistan these proportions were 72.1% [64], 63.5% [63], and 62.1% [62] among MSWs, and 83.7% [64], 77.8% [63], and 59.2% [62] among HSWs.

## Discussion

### Status of the Evidence

Contrary to widely held perceptions of very limited data on HIV among MSM in MENA, the systematic review undertaken in this work shows that there is considerable and increasing epidemiological evidence on HIV and risk behavior among MSM in this region. The evidence nevertheless is highly heterogeneous in terms of quality, and HIV research capacity varies substantially from one country to another. Several countries, such as Egypt, Iran, Lebanon, Morocco, Pakistan, Sudan, and Tunisia, have made significant strides in building their capacity within the last few years, and there has been steady improvement in the quality of HIV data among MSM. Earlier studies suffered from methodological limitations and/or were mostly descriptive and qualitative in nature, such as formative assessments. A fraction of data originated from facility- or venue-based surveillance on convenient samples of MSM and, results therefore were not necessarily representative.

A shift in nature of evidence among MSM materialized in the last few years with the implementation of several integrated biobehavioral surveillance surveys incorporating state-of-the-art sampling methodologies for hidden and hard-to-reach populations, such as respondent-driven sampling (RDS) (Table 3). However, recent sampling designs still appear to be biased towards visible MSM populations such as MSWs and may not provide a fully representative portrayal of the diverse typologies of MSM in this region. This is exacerbated by the small sample size in some of these studies where the RDS was not able to reach the desired sample size in several settings.

### Trend of Emerging Epidemics among MSM in MENA

**Figure 2:**
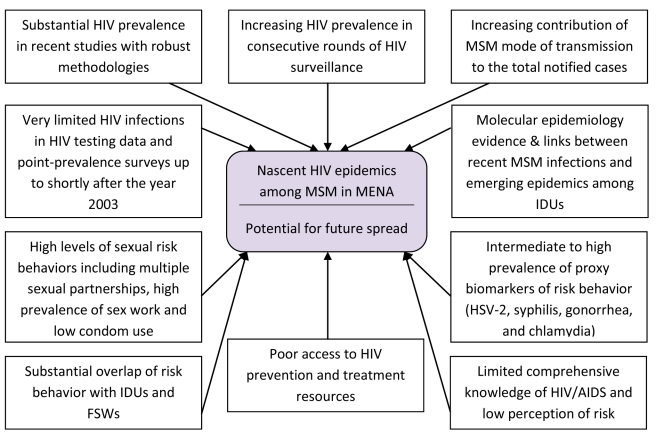
Data synthesis. Summary of the synthesis and triangulation of biological, behavioral, and contextual data about HIV among MSM in MENA corroborating emerging epidemics and HIV epidemic potential.

**Figure 3:**
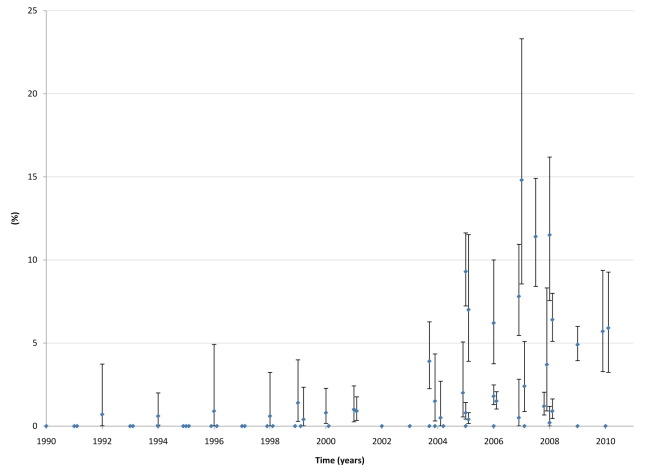
HIV prevalence among MSM in MENA, 1990–2010. This graph displays available HIV point-prevalence measures (from Tables 3 and S2) and 95% confidence intervals among MSM in all MENA countries with available measures, irrespective of study methodology. Studies with sample size of less than 100 were excluded because of very wide confidence intervals. Countries with available data include Egypt, Iran, Iraq, Jordan, Lebanon, Morocco, Pakistan, Sudan, Syria, and Tunisia. Each blue dot represents one HIV prevalence measure among MSM from one of the listed MENA countries for the specific year, while the bars around it define the limits of the 95% confidence interval around the prevalence measure. The graph indicates limited transmission until after 2003, when considerable HIV prevalence started to be apparent in most studies and surveys.

Figure 2 summarizes the synthesis and triangulation of the entirety of the data presented above and that corroborate the main findings of this analysis, namely that there appears to be nascent HIV epidemics among MSM in MENA and that there is potential for further HIV spread among this population group in this region. In consideration of the same conclusion gleaned independently from each of the several lines of direct and indirect evidence delineated in this article, it appears that the majority of the observed epidemics are recent, and did not reach concentration (HIV prevalence >5% [99]) before the year 2003, rather than long-standing epidemics (Figure 3). The evidence indicating this conclusion includes a considerable HIV prevalence documented in recent well-designed studies and venue-based surveillance after limited or no prevalence in earlier years (Tables 3 and S2; Figure 3); the increasing HIV prevalence suggested by some of the best-designed studies (Table 3); the rising contribution of MSM transmission in case notification reports (Table 2); and the epidemiological and phylogenetic evidence linking, in some settings (mainly Iran and Pakistan), MSM transmission to recent IDU epidemics [85]–[88],[100].

It might be argued that part of the apparent recent increase in HIV prevalence can be explained by improvements in study design and surveillance methodologies, as an element of uncertainty about the representativeness of earlier point-prevalence measures cannot be discarded. This is suggested by the data from Egypt, for example, where the 2006 case notification report documented zero infections among 1,471 MSM tested, at a time when an RDS study documented a 6.2% HIV prevalence during that same year. However, it is worth noting that populations with the highest risk behavior and exposure to HIV are usually the most visible, such as MSWs, and tend to be oversampled in convenience sampling methodologies versus the probability-based RDS sampling technique, which is more likely to reach hidden populations that practice less-risky behaviors [26],[101]. The fact that the observed HIV prevalence in MENA is increasing in recent studies with robust sampling designs such as RDS further suggest credence to the thesis of rising epidemics among MSM in MENA.

Moreover, while HIV testing reports and earlier surveys might not be capturing fully the epidemic dynamics among MSM, the recent HIV prevalence measures—irrespective of their methodology and the totality of data synthesized in this article—complemented by scattered anecdotal evidence from different sources and testimonies by field workers, medical practitioners, NGO members, and public health officials, all suggest that HIV epidemic transmission among MSM in most of MENA countries is a recent phenomenon that has become evident only in the last few years. The inter-setting variability in prevalence among samples within the same country, such as in Pakistan and Tunisia [13],[62]–[64], further alludes to the possibility of hidden subepidemics in other settings or other countries in MENA, and that HIV may not have yet expanded its spread and reached its epidemic potential in the different parts of each country.

It is also worth noting that, given that male same-sex mode of transmission carries the highest stigma in MENA, the reported proportions of MSM transmissions in case notification reports may underestimate the true contribution of MSM transmission to the burden of HIV infection. In a substantial fraction of HIV infections, the mode of transmission continues to be characterized as unknown such as in Algeria, Lebanon, and Saudi Arabia, where 24% [102] and 27% [103], 23% [59], and 29% [104] of HIV infections, respectively, are diagnosed with no identifiable risk behaviors. It is plausible that MSM transmission explains part of these uncharacterized HIV infections and that the rising contribution of MSM mode of transmission to the total notified cases might be even more pronounced than what is reported.

A trend of emerging HIV epidemics among MSM has been observed in other countries beyond MENA but with similar sociocultural background such as in Indonesia. In this Muslim majority country, HIV prevalence was nil among MSM until the end of 1993; but a survey in 2002 reported an HIV prevalence of 22.0% among transgender sex workers, 3.6% among MSWs, and 2.5% among self-identified MSM [105]. These observations accompany a trend in which HIV prevalence among MSM has increased rapidly during this decade in many countries with different sociocultural contexts, particularly in Asia and to some extent in Latin America [26],[106]. For example in Beijing, China, three consecutive RDS surveys documented an increasing HIV prevalence among MSM from 0.4% in 2004, to 4.6% in 2005, to 5.8% in 2006 [107].

### HIV Epidemic Potential among MSM

Several factors suggest the possibility of further expansion of HIV epidemics among MSM in MENA (Figure 2), and the observed low HIV prevalence among certain MSM groups should not be necessarily interpreted as limited potential for future spread. As described above, MSM in MENA engage in considerable levels of sexual risk behaviors. Multiple sexual partnerships of different kinds are practiced by the majority of MSM; commercial sex work including selling and paying for sex is prevalent; condom and lubricant use is limited; and overlap with opposite-sex sex and injecting drug risk behaviors is substantial.

The appreciable prevalence of proxy biological markers of sexual risk behavior, such as herpes simplex virus type 2 (HSV-2), syphilis, gonorrhea, and chlamydia, is another indicator of potential HIV spread among certain MSM groups in MENA. For example, the prevalence of HSV-2 among MSM in Turkey was reported to be 26% [108], and in Pakistan HSV-2 prevalence ranged between 2.5% and 54.0% among different MSW populations [39]. Bacterial STIs were also found to be prevalent among MSM in MENA. In Egypt, 7.5% of MSM surveyed in one study had antibodies for syphilis, 8.8% had gonorrhea, and the same proportion had chlamydia [72]. The prevalence measures of these bacterial infections were even more substantial among different groups of *hijras* and MSWs in Pakistan, where the prevalence of syphilis reached up to 60% [109], of gonorrhea up to 20% [39], and of chlamydia up to 29% [109].

Although male circumcision is nearly universal in MENA [110], and has been associated with a 60% efficacy against HIV acquisition in heterosexual sex [111]–[113], the evidence for a protective effect during anal sex remains insufficient, weak, and inconsistent [114]. Therefore, male circumcision may not be a protective factor for MSM in MENA.

### HIV Prevention Interventions among MSM in MENA

The window of opportunity for prevention of further HIV transmission among MSM is narrowing, and prompt action and robust interventions are needed. Interventions at this stage need to be prioritized for the most vulnerable MSM subpopulations such as MSWs and transgender people who, in MENA as in other regions [26],[54], appear to be at the highest risk of HIV exposure. It is also essential to expand scientific research on MSM through the establishment of surveillance systems using state of the art sampling methodologies of hard-to-reach populations. A better understanding of the MSM community and subpopulations; their ethnographies and typologies; their social behavior with each other, their family members, and the society at large; their sexual practices and networks; and their population size estimates, is also warranted.

Coverage of HIV interventions among MSM in this region continues to be limited, with poor access to testing, prevention, and treatment services. An example of this inadequate response is the level of HIV testing among this population group. Studies have documented that 2.0%–22.1% of MSM in Egypt [71],[97], 42.5% in Iran [27], 32.0% in Jordan [80], 22.0% in Lebanon [83], 1.0%–14.8% (MSWs) and 5.9%–23.4% (HSWs) in Pakistan [39],[62]–[63], 20.2% in Sudan [69], and 21.5% in Tunisia [13], have ever tested for HIV.

Promising efforts, though, have materialized in recent years, and several countries initiated outreach programs for prevention of HIV transmission among MSM [115]. Most fruitful programs have been those involving governments working with NGOs despite the challenging social, political, and legal climate such as in Algeria, Lebanon, Morocco, Pakistan, and Tunisia [27],[116]. These programs included HIV counseling and testing, provision of condoms and lubricants, STI services, prevention information, outreach peer education, and programs addressing legal, psychological, and social needs of MSM [27],[116]–[121].

The availability of HIV services is not the only barrier to the coverage of HIV interventions among this population group. MSM may not be aware of existing services within their communities, and even if they are aware of such programs, service utilization is far below what is desirable. Pakistan provides an example to illustrate this challenge. Only up to 13.6% of MSWs and up to 30.6% of HSWs were aware of HIV prevention programs in their city, and much smaller proportions of 2.5%–18.3% ever used them [62]–[64]. Despite these limitations, the programs have been successful in achieving risk behavior reduction among those who accessed them. Consistent condom use was reported by 24.6% [63] and 52.7% [62] of MSWs who used the programs, but only by 7.5% [63] and 36.5% [62] of MSWs who never used them.

Prevention of male-to-male HIV transmission must be set as a top priority for HIV/AIDS strategies in MENA, and obstacles must be addressed for the provision of comprehensive sexual health care for MSM [92]. Considering the sensitivity of working with this stigmatized population, creative mechanisms need to be developed for working with MSM even if discreetly. In addition to the key role of NGOs that have already proved effective in reaching out to MSM in many settings, a successful formula for HIV efforts could be for governments or other organizations to fund and support the establishment of NGOs that would provide services to MSM. NGOs may enable governments to deal with MSM indirectly, thereby avoiding political or cultural sensitivities in explicit outreach efforts [122]–[123].

Given the specter of expanding epidemics among MSM, and the sociocultural and political context of this region, it is probably best if this public health issue is approached from a public health perspective by disentangling it from the ongoing acrimonious debate and cynical calculus of power politics about MSM civil rights and liberties in MENA. This approach, however, should not be seen as contradictory to the concepts of rights and enabling environments, but as an effective strategy for HIV prevention among MSM within the MENA context. Despite the focus on public health, this approach must encompass basic rights such as security guarantees that are essential for the administration of interventions for this community and for the protection of MSM from barriers that may hinder accessibility of such services.

### Study Limitations

One of the limitations of the present study is that there are limited longitudinal data among MSM in MENA that are derived from the same population and that use the same standardized sampling methodology and study protocols to facilitate conclusive trend analyses. Another limitation is that the quality and quantity of epidemiological evidence varies from one country to another and our analyses could not incorporate sufficient representative evidence from a number of MENA countries. The nature of HIV epidemiology among MSM may differ from one MENA country to another as the region is diverse, despite the underlying similarities in sociocultural contexts. Moreover, some of the available studies might be biased by sampling the most visible part of MSM populations, such as MSWs, and may not be representative of the MSM population at large. Some of the studies in the region also do not provide sufficient details about the methodology, sampling techniques, and other technical information, thereby limiting our ability to conduct detailed and specific inferences concerning such data.

To address these limitations, we used in our analyses a triangulation approach in which we incorporated and analyzed multiple data sources and data types covering the diverse epidemiological aspects of HIV epidemic transmission. We also highlighted heterogeneities in the region where necessary. For example, although the main longitudinal data in the region are from Pakistan and more recently from Egypt, longitudinal data are only one of several lines of evidence that were synthesized to support our thesis. The several distinct lines of evidence, whether biological, behavioral, or contextual, are all consistent in drawing a picture indicating the emergence of nascent HIV epidemics among MSM in MENA (Figure 2).

### Conclusion

The present study is the first review of its kind in MENA and includes the largest body of evidence on HIV and MSM in this region. Contrary to most review articles, which provide analyses on already published data in the scientific literature, this comprehensive systematic review presents a large volume of data, most of which is appearing to our knowledge for the first time in the scientific literature.

The synthesis and analysis of the epidemiological data reviewed indicates that HIV prevalence among MSM in MENA appears to be at lower levels than among MSM in most other settings around the globe [2],[26]. The contribution of MSM transmissions to all diagnosed HIV transmissions appears also to be substantially lower than that in other settings where MSM transmission plays the key role in HIV epidemic dynamics, such as in Asia, Europe, Latin America, North America, and the Pacific [2],[106]. However, after two decades of apparently limited HIV transmission among MSM, HIV prevalence and the proportion of transmissions among MSM appear to be increasing in MENA. The very low HIV prevalence still found in some MSM populations is more likely than not to reflect a recent or lack of virus introduction into these populations, possibly due to their isolation rather than lack of epidemic potential, as suggested by the high risk and vulnerability context described above. When the HIV virus found its way into some of these populations and their sexual networks, indigenous chains of HIV transmission emerged leading, in some cases, to concentrated HIV epidemics.

The epidemiologic evidence delineated throughout this article, and the rather limited HIV prevalence in MENA in all of risk groups apart from MSM and IDUs [27],[124], lay out the causal logic that underpins our hypothesis of emerging HIV epidemics among MSM in MENA and suggests that MSM could materialize as the pivotal risk group for HIV sexual transmission in this region in the next decade.

## Supporting Information

Table S1 Contribution of MSM mode of transmission to the total diagnosed HIV/AIDS cases by country as per various studies/reports.
(0.07 MB DOC)

Table S2 HIV point-prevalence measures from voluntary counselling and testing (VCT) surveys, sentinel surveillance, various surveys with non-specific methodologies, and rate of HIV positive testing among MSM in MENA over the years.
(0.11 MB DOC)

Table S3 Measures of sexual risk behavior among MSM including MSWs and HSWs in MENA.
(0.17 MB DOC)

Table S4 Condom use among different MSM populations in MENA.
(0.19 MB DOC)

Table S5 Measures of overlapping MSM and IDU risk behaviors in MENA.
(0.12 MB DOC)

Table S6 Description of various sampling methodologies used in surveying MSM in MENA.
(0.04 MB DOC)

Text S1 PRISMA checklist.
(0.07 MB DOC)

Text S2 Details of data sources and search criteria.
(0.06 MB DOC)

Text S3 List of MENA countries.
(0.04 MB DOC)

## Abbreviations

FSW: female sex worker
HCV: hepatitis C virus
HSV-2: herpes simplex virus type 2
HSW: *hijra* sex worker
IDU: injecting drugs user
MENA: Middle East and North Africa
MSM: men who have sex with men
MSW: male sex worker
RDS: respondent driven sampling
STI: sexually transmitted infection
