# Supplementary material for: Are HIV Epidemics among Men Who Have Sex with Men Emerging in the Middle East and North Africa?: A Systematic Review and Data Synthesis
Source: PLoS Med. 2011 Aug 2;8(8):e1000444. doi: 10.1371/journal.pmed.1000444 (PMC3149074; doi:10.1371/journal.pmed.1000444)
Supplement: Table S1 — Contribution of MSM mode of transmission to the total diagnosed HIV/AIDS cases by country as per various studies/reports. (0.07 MB DOC) [file pmed.1000444.s001.doc]

**Table S1.** Contribution of MSM mode of transmission to the total diagnosed HIV/AIDS cases by country as per various studies/reports.

| **Country** | **Proportion of the total HIV/AIDS cases due to MSM transmission** |
| --- | --- |
| Algeria | 4% [1], 4.7% [2] |
| Egypt | 20% [3], 25.5% [4] |
| Lebanon | 15.1% [5] |
| Morocco | 7% [6] |
| Pakistan | 3.2% [7], 7% [8-9], 10% [10] |
| Palestine | 1% [11] |
| Saudi Arabia | 2.5% [12], 4.9% [13] |
| Syria | 8% [14], 9.2% [15] |
| Tunisia | 4% [16], 6.5%^§^ [17], 9%^*^ [18] |
| Yemen | 16% [19] |

This table summarizes the proportions of the total diagnosed HIV/AIDS cases in various populations in MENA that is due to anal sex between males, as reported by various studies and reports. The denominator in these figures consists of the total number of HIV/AIDS cases in a given populations (males and females) and the numerator is the number of these cases which was due to male same-sex sex.

^*^ The denominator consisted of only *men* infected with HIV.

**References**

1. Alem A, Boudjadja S, Cherrouf A (1999) Données épidémiologiques sur l'infection par le VIH en Algérie (French) [Epidemiologic data of HIV infection in Algeria]. Archives de l'Institut Pasteur d'Algérie 63: 21-35.

2. Fares EG FY (2005) Rapport de l’Algérie sur le suivi en 2005 de la déclaration d’engagement de la session extraordinaire de l’assemblée générale des nations unies sur le VIH/Sida (UNGASS) (French) [The United Nations General Assembly Special Session on HIV/AIDS (UNGASS) 2005 progress report - Algeria]. Alger, Algeria.

3. UNAIDS/WHO (2005) AIDS epidemic update 2005. Geneva, Switzerland.

4. Aly MM (1999) Pulmonary manifestations in cases of acquired immune deficiency syndrome in Egypt : Retrospective study. Cairo, Egypt: Cairo University.

5. Nakib ME (2008) National HIV/AIDS database. Lebanon National AIDS Control Programme, Beirut, Lebanon.

6. Elharti E (2002) HIV epidemiology in Morocco: a nine-year survey (1991–1999). ElHarti E, personal communication, updated data up to 2008. International journal of STD and AIDS 13: 839-842.

7. Khan OA, Hyder AA (1998) HIV / AIDS among men who have sex with men in Pakistan. Sex Health Exch: 12-13, 15.

8. UNAIDS/WHO (2006) AIDS epidemic update 2006. Geneva, Switzerland.

9. Rajabali A, Khan S, Warraich HJ, Khanani MR, Ali SH (2008) HIV and homosexuality in Pakistan. Lancet Infect Dis 8: 511-515.

10. Iqbal J, Rehan N (1996) Sero-prevalence of HIV: six years' experience at Shaikh Zayed Hospital, Lahore. J Pak Med Assoc 46: 255-258.

11. UNAIDS (2007) Key Findings on HIV Status in the West Bank and Gaza. Working Document. UNAIDS Regional Support Team For the Middle East and North Africa. Cairo, Egypt.

12. Madani TA, Al-Mazrou YY, Al-Jeffri MH, Al Huzaim NS (2004) Epidemiology of the human immunodeficiency virus in Saudi Arabia; 18-year surveillance results and prevention from an Islamic perspective. BMC Infect Dis 4: 25.

13. Alrajhi AA, Halim MA, Al-Abdely HM (2004) Mode of transmission of HIV-1 in Saudi Arabia. Aids 18: 1478-1480.

14. Syria National AIDS Programme (2004) HIV/AIDS Female Sex Workers KABP Survey in Syria. Damascus, Syria

15. Syria National HIV/AIDS program (2004) National HIV/AIDS database. Syria Ministry of Health.

16. Programme National de Lutte Contre le Sida, Ministere de la Sante en Tunisie (March 2005) Analyse de la situation et de la réponse au VIH/SIDA en Tunisie (French) [Analysis of the situation and response to HIV/AIDS in Tunisia]. Tunis, Tunisia.

17. Hsairi M, Ben Abdallah S (2007) Analyse de la situation de vulnérabilité vis-à-vis de l’infection à VIH des hommes ayant des relations sexuelles avec des hommes. Rapport Final, version abrégée (French) [Analysis of the HIV vulnerability settings of men who have sex with men. Final report, short version]. Tunisia Ministry of Health, Tunis, Tunisia.

18. Tammam A (2010) Surveillance of HIV prevention among drug users in Tunisia. Abstract no THPE0498 AIDS 2010 - XVIII International AIDS Conference. Vienna, Austria

19. Lambert L (2007) HIV and development challenges in Yemen: which grows fastest? Health Policy and Planning 22: 60.
