# Supplementary material for: Are HIV Epidemics among Men Who Have Sex with Men Emerging in the Middle East and North Africa?: A Systematic Review and Data Synthesis
Source: PLoS Med. 2011 Aug 2;8(8):e1000444. doi: 10.1371/journal.pmed.1000444 (PMC3149074; doi:10.1371/journal.pmed.1000444)
Supplement: Table S2 — HIV point-prevalence measures from voluntary counselling and testing (VCT) surveys, sentinel surveillance, various surveys with non-specific methodologies, and rate of HIV positive testing among MSM in MENA over the years. (0.11 MB DOC) [file pmed.1000444.s002.doc]

**Table S2.** HIV point-prevalence measures from VCT surveys, sentinel surveillance, various surveys with non-specific methodologies, and rate of HIV positive testing among MSM in MENA over the years.

| **Year** | **Djibouti** | **Egypt** | **Iran** | **Iraq** | **Jordan** | **Kuwait** | **Lebanon** | **Morocco** | **Pakistan** | **Sudan** | **Syria** | **Tunisia** | **Yemen** |
| --- | --- | --- | --- | --- | --- | --- | --- | --- | --- | --- | --- | --- | --- |
|  | N (%) | N (%) | N (%) | N (%) | N (%) | N (%) | N (%) | N (%) | N (%) | N (%) | N (%) | N (%) | N (%) |
| **1986** |  | (0.0) |  |  |  |  |  |  |  |  |  |  |  |
| **1989** |  |  |  |  |  | 21 (0.0) |  | 55 (10.9) |  | 52 (7.7) | 32 (0.0) | 72 (0.0) |  |
| **1990** | 30 (9.7) |  | 300 (0.0) |  |  | 2 (0.0) |  |  |  |  | 2 (0.0) |  |  |
| **1991** |  | 110 (0.0) | 638 (0.0) |  |  |  |  |  | 37 (0.0) |  |  |  |  |
| **1992** |  | 47 (2.1) | 36 (0.0) | 40 (0.0) |  | 41 (0.0) |  |  |  |  | 90 (0.0) |  |  |
|  |  | 147 (0.7) |  |  |  |  |  |  |  |  |  |  |  |
| **1993** |  | 117 (0.0) |  | 280 (0.0) | 23 (0.0) |  |  |  | 61 (1.6) |  | 22 (9.1) |  |  |
| **1994** |  | 118 (0.0) | 47 (0.0) | 234 (0.0) |  |  | 17 (0.0) |  | 2 (0.0) |  | 359 (0.6) |  |  |
| **1995** |  | 110 (0.0) |  | 559 (0.0) | 2 (0.0) |  | 4 (0.0) |  | 439 (0.0) |  | 55 (3.6) |  | 4 (25.0) |
| **1996** |  | 32 (3.1) |  | 172 (0.0) |  |  |  |  | 17 (0.0) |  | 111 (0.9) |  |  |
|  |  | 132 (0.0) |  |  |  |  |  |  |  |  |  |  |  |
| **1997** |  | 140 (0.0) |  | 78 (0.0) |  |  |  |  | 14 (7.1) |  | 198 (0.0) |  |  |
| **1998** |  | 52 (0.0) |  | 354 (0.0) |  |  |  |  |  |  | 170 (0.6) |  | 41 (0.0) |
|  |  | 152 (1.3) |  |  |  |  |  |  |  |  |  |  |  |
| **1999** |  | 236 (0.4) |  |  |  |  |  |  | 15 (0.0) |  | 372 (0.0) |  | 76 (0.0) |
|  |  | 217 (1.4) |  |  |  |  |  |  |  |  | 192 (0.0) |  |  |
| **2000** |  | 382 (0.8) |  |  |  |  |  |  | 4 (50.0) |  | 160 (0.0) |  | 15 (0.0) |
| **2001** |  | 815 (0.9) |  |  |  |  |  |  | 9 (11.1) |  | 69 (0.0) |  | 1 (100) |
|  |  | 420 (1.0) |  |  |  |  |  |  |  |  |  |  |  |
| **2002** |  | 470 (0.0) |  |  |  |  |  |  | 4 (0.0) |  | 62 (0.0) |  |  |
| **2003** |  | 613 (0.0) |  |  |  |  |  |  |  |  | 37 (0.0) |  |  |
| **2004** |  | 632 (0.0) |  |  |  |  |  |  |  |  | 18 (0.0) |  |  |
|  |  | 557 (0.0) |  |  |  |  |  |  |  |  |  |  |  |
| **2005** |  | 528 (0.0) |  |  |  |  |  |  |  |  | 56 (0.0) |  |  |
| **2006** |  | 1,471 (0.0) |  |  |  |  |  |  |  |  | 188 (0.0) |  | 27 (0.0) |
| **2007** |  |  |  |  |  |  |  |  |  |  | 9 (0.0) |  |  |
| **2008** |  | 229 (11.5) |  |  |  |  |  |  |  |  |  |  |  |
|  |  |  |  |  |  |  |  | 1,147 (1.2) |  |  |  |  |  |
| **2009** |  |  |  |  |  |  |  | 39 (0.0) |  |  |  |  |  |
|  |  |  |  |  |  |  |  | 1,216 (2.2) |  |  |  |  |  |

This table summarizes data extracted from country-level databases [1-3], conference proceedings [4], UNAIDS epidemiological fact sheets [5], US Census Bureau database of HIV/AIDS [6], and the WHO/EMRO HIV testing database [7]. Most of the data in this table are from earlier years and the methodology/quality of the point-prevalence measures is not well-defined. Cells display the number of MSM that were tested and the percentage of positive cases among them.

**References**
