# Supplementary material for: Are HIV Epidemics among Men Who Have Sex with Men Emerging in the Middle East and North Africa?: A Systematic Review and Data Synthesis
Source: PLoS Med. 2011 Aug 2;8(8):e1000444. doi: 10.1371/journal.pmed.1000444 (PMC3149074; doi:10.1371/journal.pmed.1000444)
Supplement: Table S3 — Measures of sexual risk behavior among MSM including MSWs and HSWs in MENA. (0.17 MB DOC) [file pmed.1000444.s003.doc]

**Table S3.** Measures of sexual risk behavior among MSM including MSWs and HSWs in MENA.

|  | **Afghanistan** | **Egypt** | **Iran** | **Jordan** | **Lebanon** | **Oman** | **Pakistan** | **Sudan** | **Tunisia** |
| --- | --- | --- | --- | --- | --- | --- | --- | --- | --- |
| **% having multiple partners** ^a^ |  | 58.1% [1], 90% [2] | 81.9% [3] |  |  |  | 4.3% [4] | 97.2% [5-6] | 90.1% [7] |
| **Average # of partners:** |  |  |  |  |  |  |  |  |  |
| In the last year |  |  |  |  | 9.5 [8] | 2 [9] | 42^b^ [10] |  |  |
| In the last 6 months |  |  |  | 4.4^c^ [11],  5^c^ [11] |  |  |  | 13^c^ [12] | 3.8^c^ [13],  14.3 [7] |
| Per week |  | 3.2^c^ [14] |  |  |  |  |  |  |  |
| **Type of sex act** ^a^ |  |  |  |  |  |  |  |  |  |
| Insertive anal sex |  | 66.7% [15],  74% [14],  82.0% [16],  88.1% [15], |  | 95.4% [11] |  |  |  |  | 84.5% [13] |
| Receptive anal sex |  | 8.8% [15],  35.9% [15],  51.0% [16],  59.3% [15],  90.6% [14] |  | 79.5% [11] |  |  |  |  | 49.0% [13] |
| Oral sex |  | 88.6% [15],  92.0% [15],  93.0% [15] |  | 89.1% [11] |  |  | 39-42% [17] | 56.2% [12], 97.6% [18] | 92.4% [13] |
| **% experienced forced anal sex:** |  |  |  |  |  |  |  |  |  |
| Ever: |  |  |  |  |  |  |  |  | 13.3% [13] |
| In last year |  | 6.3% [16] | 47.8% [3] |  | 2% [19] |  | 40% [17] | 63.7% [12] | 8.8% [13],  26.4% [7] |
| In the last 6 months |  | 6.4% [15],  12.7%[15],  14.6% [15] |  |  |  |  |  |  |  |
| **Type of partnership** ^a^ |  |  |  |  |  |  |  |  |  |
| Steady |  |  | 61.2% [3] |  | 74.3% [8],  86% [19] |  |  |  |  |
| Casual |  |  | 82.2% [3] |  | 51.8% [8] |  |  |  | 90.8% [7] |
| Commercial |  | 31.8% [15],  33.2% [15],  92.0% [15] | 53.8% [3] | 65.1% [11] | 54.5% [8] |  | 15% [17] | 80.3% [18] | 12% [7] |
| **Male sex work** |  |  |  |  |  |  |  |  |  |
| Prevalence among MSM ^a^ |  | 20% [20], 42.0% [16] | 28.6%[21] |  | 27% [19],  36% [19] |  | 84% [17],  96% [22],  99% [17] | 67.4% [12], 75.5% [6] |  |
| Average # of clients per: |  |  |  |  |  |  |  |  |  |
| Working day | 3 [23] |  |  |  |  |  | 1.9-2.1^d^ [24-26],  2.5-2.6^e^ [24-26] |  |  |
| Month |  |  |  |  |  |  | 20.3-31.1^d^ [24-26],  32.0-49.1^e^ [24-26] |  |  |
| The last 6 months |  |  |  |  |  |  |  | 23.9^c^ [12] |  |
| % had sex with non-commercial partners in the last month |  |  |  |  |  |  | 21.1-44.0%^e^ [24-26],  31.8-37.7%^d^ [24-26], 40% (*hijras*) [17] |  |  |

This table summarizes data on measures of sexual risk behavior among MSM as reported by various studies in MENA countries. It also summarizes the prevalence of male sex work and related practices in MENA.

^a^ Some may reflect different time frames

^b^ Population included Pakistan, India, and Bangladesh

^c^ Minimum average number of partners calculated based on available data

^d^ Range of numbers observed among MSWs in the three rounds of surveillance

^e^ Range of numbers observed among HSWs in the three rounds of surveillance

**References**

1. Abdel-Rahman I, Soliman C, Bahaa T, Moustafa M, Shawky S, et al. (2010) MSM access to VCT in a conservative environment, case of Egypt. Abstract no WEPE0318 AIDS 2010 - XVIII International AIDS Conference. Vienna, Austria.

2. El-Sayed N, Abdallah M, Abdel Mobdy A, Abdel Sattar A, Aoun E, et al. (2002) Evaluation of Selected Reproductive Health Infections in Various Egyptian Population Groups in Greater Cairo, MOHP, IMPACT/FHI/USAID. Cairo, Egypt.

3. Abu-Raddad L, Akala FA, Semini I, Riedner G, Wilson D, et al. (2010) Characterizing the HIV/AIDS epidemic in the Middle East and North Africa: Time for Strategic Action. Middle East and North Africa HIV/AIDS Epidemiology Synthesis Project. World Bank/UNAIDS/WHO Publication. Washington DC: The World Bank Press.

4. Khanani MR, Somani M, Khan S, Naseeb S, Ali SH (2010) Prevalence of single, double, and triple infections of HIV, HCV and HBV among the MSM community in Pakistan. J Infect 61: 507-509.

5. Anonymous (2007) Improving HIV/AIDS Response among Most at Risk Population in Sudan. Orientation Workshop, 16^th^ April 2007.

6. Elrashied S (2006) Prevalence, knowledge and related risky sexual behaviours of HIV/AIDS among receptive men who have sex with men (MSM) in Khartoum State, Sudan, 2005. Abstract TUPE0509. AIDS 2006 - XVI International AIDS Conference. Toronto, Canada

7. Hsairi M, Ben Abdallah S (2007) Analyse de la situation de vulnérabilité vis-à-vis de l’infection à VIH des hommes ayant des relations sexuelles avec des hommes. Rapport Final, version abrégée (French) [Analysis of the HIV vulnerability settings of men who have sex with men. Final report, short version]. Tunisia Ministry of Health, Tunis, Tunisia.

8. Hermez J, Aaraj E, Dewachi O, Chemaly N HIV/AIDS prevention among vulnerable groups in Beirut, Lebanon. Powerpoint presentation. Lebanon National AIDS Control Program, Beirut, Lebanon.

9. Oman Ministry of Health (2006) HIV Risk among Heroin and Injecting Drug Users in Muscat, Oman. Quantitative Survey. Preliminary Data. Muscat, Oman.

10. Khan OA, Hyder AA (1998) HIV / AIDS among men who have sex with men in Pakistan. Sex Health Exch: 12-13, 15.

11. Jordan National AIDS Program (2010) Preliminary analysis of Jordan IBBSS among MSM. Ministry of Health, Jordan.

12. Elrashied SM (2006) Generating Strategic Information and assessing HIV/AIDS Knowledge, Attitude and Behaviour and Practices as well as Prevalence of HIV1 among MSM in Khartoum State, 2005. A draft report submitted to Sudan National AIDS Control Programme. Together Against AIDS Organization (TAG). Khartoum, Sudan.

13. Ministere de la Sante Publique en Tunisie, Association Tunisienne de Lutte Contre les MST et le SIDA (2010) Enquête sérocomportementale auprès des hommes ayant des rapports sexuels avec des hommes en Tunisie (French) [Biobehavioral survey among men who have sex with men in Tunisia]. Tunis, Tunisia.

14. El-Sayyed N, Kabbash IA, El-Gueniedy M (2008) Risk behaviours for HIV/AIDS infection among men who have sex with men in Cairo, Egypt. East Mediterr Health J 14: 905-915.

15. Egypt Ministry of Health and Population National AIDS Program (2010) HIV/AIDS biological and behavioral surveillance survey, Round II, Summary report Egypt 2010. Cairo, Egypt.

16. Egypt Ministry of Health and Population National AIDS Program (2006) HIV/AIDS biological and behavioral surveillance survey, Round I, Summary report Egypt 2006. Cairo, Egypt.

17. Khan AA, Rehan N, Qayyum K, Khan A (2008) Correlates and prevalence of HIV and sexually transmitted infections among Hijras (male transgenders) in Pakistan. Int J STD AIDS 19: 817-820.

18. Elrashied S (2008) HIV sero-prevalence and related risky sexual beahviours among insertive men having sex with men (IMSM) in Khartoum state, Sudan, 2007. AIDS 2008 - XVII International AIDS Conference. Mexico city, Mexico.

19. Mahfoud Z, Afifi R, Ramia S, El Khoury D, Kassak K, et al. (2010) HIV/AIDS among female sex workers, injecting drug users and men who have sex with men in Lebanon: results of the first biobehavioral surveys. AIDS 24 Suppl 2: S45-54.

20. El-Sayed N, Darwish A, El-Geeneidy M, Mehrez M (1994) Knowledge, Attitude, and Practice of Homosexuals Regarding HIV in Egypt. National AIDS Program, Ministry of Health and Population, Cairo, Egypt.

21. Eftekhar M, Feizzadeh A, Moshtagh Bidokhti N, Setayesh H, Vasigh A, et al. (2008) High risk behavior and HIV/AIDS prevalence among men having sex with men: the first report from Iran. AIDS 2008 - XVII International AIDS Conference. Mexico City, Mexico.

22. Bokhari A, Nizamani NM, Jackson DJ, Rehan NE, Rahman M, et al. (2007) HIV risk in Karachi and Lahore, Pakistan: an emerging epidemic in injecting and commercial sex networks. Int J STD AIDS 18: 486-492.

23. SAR AIDS, The World Bank (2008) Mapping and Situation Assessment of Key Populations at High Risk of HIV in Three Cities of Afghanistan. Human Development Sector, South Asia Region, World Bank. Washington DC, USA.

24. Pakistan National AIDS Control Program (2005) HIV Second Generation Surveillance In Pakistan. National Report Round I. Canada-Pakistan HIV/AIDS Surveillance Project. National Aids Control Program, Ministry Of Health, Pakistan.

25. Pakistan National AIDS Control Program (2006-07) HIV Second Generation Surveillance In Pakistan. National Report Round II. Canada-Pakistan HIV/AIDS Surveillance Project. National Aids Control Program, Ministry Of Health, Pakistan.

26. Pakistan National AIDS Control Program (2008) HIV Second Generation Surveillance In Pakistan. National Report Round III. Canada-Pakistan HIV/AIDS Surveillance Project. National Aids Control Program, Ministry Of Health, Pakistan.
