# Supplementary material for: Are HIV Epidemics among Men Who Have Sex with Men Emerging in the Middle East and North Africa?: A Systematic Review and Data Synthesis
Source: PLoS Med. 2011 Aug 2;8(8):e1000444. doi: 10.1371/journal.pmed.1000444 (PMC3149074; doi:10.1371/journal.pmed.1000444)
Supplement: Table S4 — Condom use among different MSM populations in MENA. (0.19 MB DOC) [file pmed.1000444.s004.doc]

**Table S4.** Condom use among different MSM populations in MENA.

| **Country** | **Time frame** | **MSM population** | **% of condom use in the specified time frame, (type of partnership)** |
| --- | --- | --- | --- |
| Egypt | Consistent: | General MSM: | 2.0% [1], 19.0% [2], 19.2% [3] |
|  | Regular: |  | 19.0% [4] |
|  | Last sex: |  | Oral: 5.1% [5], 11.7% [5], 14.4% [5]  Commercial: 9.2% [6], 10.7% [5], 21.9% [5], 54.9% [5]  Non-commercial: 11.8% [5], 12.7% [6], 19.2% [5], 25.0% [5] |
|  | Last six months: |  | 27.3% [7] |
|  | Ever use: |  | 21.0% [1], 47.0% [2], 47.9% [3] |
| Iran | Last sex: | General MSM: | 19.4% (with steady partner) [8], 59.5% (commercial) [8], 59.9% (with casual partner) [8] |
| Jordan | Last sex | General MSM: | 37.2% (non-commercial) [9], 60.8% (commercial) [9] |
|  | Consistent last 6 months |  | 18.9% (non-commercial) [9], 36.1% (commercial) [9] |
| Lebanon | Consistent: | General MSM: | With regular partners: 47.1% [10-11], 63% (non-commercial) [12], 73% (commercial) [12]  With non-commercial casual partners: 39% [12], 54.5% [10-11], |
|  | Last sex: |  | With regular partner: 46% (non-commercial) [12], 70% (commercial) [12]  With casual partners: 67% (non-commercial) [12] |
|  | Ever use: |  | 78.8% [13] |
| Oman | Last year: | General MSM: | 68-100% (with MSM IDUs) [14] |
| Pakistan | Consistent: | General MSM: | 3.1% (commercial) [15], 4.0% (with *hijras*^*^) [15] |
|  | Consistent last month: | MSWs: | 7.1% [16], 8.0% [17], 24.0% (commercial) [18], 22.2% (non-commercial) [18] |
|  |  | HSWs: | 5.6% [17], 7.5% [16], 19.7% (commercial) [18], 19.6% (non-commercial) [18] |
|  |  | *Banthas*^*^: | 10.0% (commercial) [19], 0.0% (with *girya*^*^) [19] |
|  |  | *Khotkis*^*^: | 8.0% (commercial) [19], 8.0% (with *girya*^*^) [19] |
|  |  | *Khusras*^*^: | 4.0% (commercial) [19], 7.0% (with *girya*^*^) [19] |
|  | Last sex: | MSWs: | 9.0% [20], 13.0% (oral) [16], 24.0% [16], 32.5% (non-commercial) [18], 34.7% (commercial) [18] |
|  |  | HSWs: | 15.0% (oral) [16], 21.0% [16], 32.3% (commercial) [18], 26.0% (non-commercial) [18] |
|  |  | *Hijras*^*^: | 12% (receptive sex; with one-time client) [21], 13% (receptive sex; with regular client) [21], 15% (with regular client) [21], 18% (with one-time client) [21] |
|  |  | *Banthas*^*^: | 23.0% (commercial) [19], 10.0% (oral) [19], 0.0% (with *girya*) [19] |
|  |  | *Khotkis*^*^: | 31.0% (commercial) [19], 8.0% (commercial oral) [19], 27.0% (with *girya*) [19] |
|  |  | *Khusras*^*^: | 25.0% (commercial) [19], 4.0% (commercial oral) [19], 7.0% (with *girya*^*^) [19] |
|  | Ever use: | General MSM: | 20% (with *hijras*^*^) [22], 20-52% (with *hijras*^*^) [23] |
| Sudan | Consistent: | Receptive MSM: | 3.3% [24] |
|  |  | Insertive MSM: | 8.8% (non-commercial) [25], 27.6% (commercial) [25] |
|  | Almost every time: |  | 50.9% [26] |
|  | Last sex: | General MSM: | 48.5% (non-commercial) [26], 58.5% (commercial) [26] |
|  |  | Receptive MSM: | 47% [24] |
|  | Last six months: |  | 89.4% (with female partners) [26] |
|  | Ever use: |  | 72.9% [26], 86.2% [24] |
| Tunisia | Consistent: | General MSM: | 19.7% (non-commercial) [27], 11.8% [28] |
|  | Last sex: |  | Non-commercial: 36.5% [28], 46.4% [27]  Commercial: 46.0% [28], 55.4% [27],  With females: 38.7% [28], 53.7% [27] |
|  | Ever use: |  | 36.2% [27] |

This table summarizes all available measures of condom use among MSM in MENA. Data is displayed by country, time-frame of use, type of MSM population, type of partnership, and type of sexual act, as warranted by the data.

^*^ *Banthas*: biological males with a male gender identity, *Giryas*: husbands of *hijras*, *Hijras and Khusras*: transgender people, *Khotkis*: Biological males who dress as men but have ‘female souls’ and feminized traits.

**References**

1. El-Sayed N, Darwish A, El-Geeneidy M, Mehrez M (1994) Knowledge, Attitude, and Practice of Homosexuals Regarding HIV in Egypt. National AIDS Program, Ministry of Health and Population, Cairo, Egypt.

2. El-Sayed N, Abdallah M, Abdel Mobdy A, Abdel Sattar A, Aoun E, et al. (2002) Evaluation of Selected Reproductive Health Infections in Various Egyptian Population Groups in Greater Cairo, MOHP, IMPACT/FHI/USAID. Cairo, Egypt.

3. El-Sayyed N, Kabbash IA, El-Gueniedy M (2008) Risk behaviours for HIV/AIDS infection among men who have sex with men in Cairo, Egypt. East Mediterr Health J 14: 905-915.

4. El-Rahman A (2004) Risky behaviours for HIV/AIDS infection among a sample of homosexuals in Cairo city, Egypt. Abstract WePeC6146. AIDS 2004 - XV International AIDS Conference. Bangkok, Thailand.

5. Egypt Ministry of Health and Population National AIDS Program (2010) HIV/AIDS biological and behavioral surveillance survey, Round II, Summary report Egypt 2010. Cairo, Egypt.

6. Egypt Ministry of Health and Population National AIDS Program (2006) HIV/AIDS biological and behavioral surveillance survey, Round I, Summary report Egypt 2006. Cairo, Egypt.

7. Abdel-Rahman I, Soliman C, Bahaa T, Moustafa M, Shawky S, et al. (2010) MSM access to VCT in a conservative environment, case of Egypt. Abstract no WEPE0318 AIDS 2010 - XVIII International AIDS Conference. Vienna, Austria.

8. Abu-Raddad L, Akala FA, Semini I, Riedner G, Wilson D, et al. (2010) Characterizing the HIV/AIDS epidemic in the Middle East and North Africa: Time for Strategic Action. Middle East and North Africa HIV/AIDS Epidemiology Synthesis Project. World Bank/UNAIDS/WHO Publication. Washington DC: The World Bank Press.

9. Jordan National AIDS Program (2010) Preliminary analysis of Jordan IBBSS among MSM. Ministry of Health, Jordan.

10. Hermez J, Aaraj E, Dewachi O, Chemaly N HIV/AIDS prevention among vulnerable groups in Beirut, Lebanon. Powerpoint presentation. Lebanon National AIDS Control Program, Beirut, Lebanon.

11. Dewachi O (2001) HIV/AIDS Prevention through outreach to vulnearable populations in Beirut, Lebanon. "Men who have sex with ohter men and HIV AIDS: a situation analysis in Beirut, Lebanon". Lebanon Ministry of Health, beirut, Lebanon. Final Report. April 29, 2001.

12. Mahfoud Z, Afifi R, Ramia S, El Khoury D, Kassak K, et al. (2010) HIV/AIDS among female sex workers, injecting drug users and men who have sex with men in Lebanon: results of the first biobehavioral surveys. AIDS 24 Suppl 2: S45-54.

13. Aaraj E Report on the situation analysis on vulnerable groups in Beirut, Lebanon. Lebanon Ministry of Health, Beirut, Lebanon.

14. Oman Ministry of Health (2006) HIV Risk among Heroin and Injecting Drug Users in Muscat, Oman. Quantitative Survey. Preliminary Data. Muscat, Oman.

15. Saleem NH, Adrien A, Razaque A (2008) Risky sexual behavior, knowledge of sexually transmitted infections and treatment utilization among a vulnerable population in Rawalpindi, Pakistan. Southeast Asian J Trop Med Public Health 39: 642-648.

16. Pakistan National AIDS Control Program (2005) HIV Second Generation Surveillance In Pakistan. National Report Round I. Canada-Pakistan HIV/AIDS Surveillance Project. National Aids Control Program, Ministry Of Health, Pakistan.

17. Pakistan National AIDS Control Program (2006-07) HIV Second Generation Surveillance In Pakistan. National Report Round II. Canada-Pakistan HIV/AIDS Surveillance Project. National Aids Control Program, Ministry Of Health, Pakistan.

18. Pakistan National AIDS Control Program (2008) HIV Second Generation Surveillance In Pakistan. National Report Round III. Canada-Pakistan HIV/AIDS Surveillance Project. National Aids Control Program, Ministry Of Health, Pakistan.

19. Hawkes S, Collumbien M, Platt L, Lalji N, Rizvi N, et al. (2009) HIV and other sexually transmitted infections among men, transgenders and women selling sex in two cities in Pakistan: a cross-sectional prevalence survey. Sex Transm Infect 85 Suppl 2: ii8-16.

20. Bokhari A, Nizamani NM, Jackson DJ, Rehan NE, Rahman M, et al. (2007) HIV risk in Karachi and Lahore, Pakistan: an emerging epidemic in injecting and commercial sex networks. Int J STD AIDS 18: 486-492.

21. Khan AA, Rehan N, Qayyum K, Khan A (2008) Correlates and prevalence of HIV and sexually transmitted infections among Hijras (male transgenders) in Pakistan. Int J STD AIDS 19: 817-820.

22. Baqi S, Shah SA, Baig MA, Mujeeb SA, Memon A (1999) Seroprevalence of HIV, HBV, and syphilis and associated risk behaviours in male transvestites (Hijras) in Karachi, Pakistan. Int J STD AIDS 10: 300-304.

23. Pakistan National AIDS Control Program (2005) Integrated Biological & Behavioral Surveillance 2004-05. Report of the Pilot Study in Karachi & Rawalpindi. Ministry of Health Canada-Pakistan HIV/AIDS Surveillance Project.

24. Elrashied S (2006) Prevalence, knowledge and related risky sexual behaviours of HIV/AIDS among receptive men who have sex with men (MSM) in Khartoum State, Sudan, 2005. Abstract TUPE0509. AIDS 2006 - XVI International AIDS Conference. Toronto, Canada

25. Elrashied S (2008) HIV sero-prevalence and related risky sexual beahviours among insertive men having sex with men (IMSM) in Khartoum state, Sudan, 2007. AIDS 2008 - XVII International AIDS Conference. Mexico city, Mexico.

26. Elrashied SM (2006) Generating Strategic Information and assessing HIV/AIDS Knowledge, Attitude and Behaviour and Practices as well as Prevalence of HIV1 among MSM in Khartoum State, 2005. A draft report submitted to Sudan National AIDS Control Programme. Together Against AIDS Organization (TAG). Khartoum, Sudan.

27. Hsairi M, Ben Abdallah S (2007) Analyse de la situation de vulnérabilité vis-à-vis de l’infection à VIH des hommes ayant des relations sexuelles avec des hommes. Rapport Final, version abrégée (French) [Analysis of the HIV vulnerability settings of men who have sex with men. Final report, short version]. Tunisia Ministry of Health, Tunis, Tunisia.

28. Ministere de la Sante Publique en Tunisie, Association Tunisienne de Lutte Contre les MST et le SIDA (2010) Enquête sérocomportementale auprès des hommes ayant des rapports sexuels avec des hommes en Tunisie (French) [Biobehavioral survey among men who have sex with men in Tunisia]. Tunis, Tunisia.
