# Supplementary material for: Are HIV Epidemics among Men Who Have Sex with Men Emerging in the Middle East and North Africa?: A Systematic Review and Data Synthesis
Source: PLoS Med. 2011 Aug 2;8(8):e1000444. doi: 10.1371/journal.pmed.1000444 (PMC3149074; doi:10.1371/journal.pmed.1000444)
Supplement: Table S5 — Measures of overlapping MSM and IDU risk behaviors in MENA. (0.12 MB DOC) [file pmed.1000444.s005.doc]

**Table S5.** Measures of overlapping MSM and IDU risk behaviors in MENA.

| **Country** | **% of injecting drug use among MSM** | **% of anal sex among male IDUs** |
| --- | --- | --- |
| Afghanistan |  | 21% [1], 23.1% [2], 25.7% [3], 32.3% [4] |
| Egypt | 2.3% [5], 4.8% [5], 4.9% [5], 6.3% [6], 10.9% [7] | 3% [8], 7.7% [5], 9.4% [7,9], 14.3% [5] |
| Iran | 53% [10] | 1.6% (IDU prisoners) [11], 5% [12], 5-17% [13-14], 6.4% [15], 7.7%[16], 11.3% [17],13.3% [18], 19.4% [19], 30% [20] |
| Lebanon | 0%[21] | 24.7% [22] |
| Morocco |  | 13.1% (74% of sample are IDUs) [23] |
| Oman |  | 12-42% [24] |
| Pakistan | Hijras: 1-2% [25], 4-17% [26]  MSWs: 0-3% [27], 4% [25], 4.2% [28], 5.0% [29], 5.2% [30], 7-9% [26]  HSWs: 5.3% [29], 6.3% [30], 4.6% [28] | 7% [31], 7.6% [32], 10.8% [33], 13.2% [30], 13.9% [28], 14% [25], 14.7% [34] [29], 47.1% [35] |
| Tunisia | 2.5% [36], 14.7% [37] |  |
| Syria |  | 5% (51% of sample are IDUs) [38] |

This table summarizes the measures of overlapping MSM and IDU risk behaviors in MENA. More specifically it displays the prevalence of injecting drug use among MSM as well as the prevalence of anal sex among male IDUs.

**References**

1. SAR AIDS, The World Bank (2008) Mapping and Situation Assessment of Key Populations at High Risk of HIV in Three Cities of Afghanistan. Human Development Sector, South Asia Region, World Bank. Washington DC, USA.

2. Nasir A, Todd CS, Stanekzai MR, Bautista CT, Botros BA, et al. (2011) Prevalence of HIV, hepatitis B and hepatitis C and associated risk behaviours amongst injecting drug users in three Afghan cities. Int J Drug Policy 22: 145-152.

3. Todd CS, Nasir A, Raza Stanekzai M, Abed AM, Strathdee SA, et al. (2010) Prevalence and correlates of syphilis and condom use among male injection drug users in four Afghan cities. Sex Transm Dis 37: 719-725.

4. C.S. Todd, A. Abed, S.A. Strathdee, B.A. Botros, N. Safi, et al. (2006) Prevalence of HIV, viral hepatitis, syphilis and risk behaviors among Injection drug users in Kabul, Afghanistan. Abstract no. TUAC0304. AIDS 2006 - XVI International AIDS Conference. Toronto, Canada.

5. Egypt Ministry of Health and Population National AIDS Program (2010) HIV/AIDS biological and behavioral surveillance survey, Round II, Summary report Egypt 2010. Cairo, Egypt.

6. Abdel-Rahman I, Soliman C, Bahaa T, Moustafa M, Shawky S, et al. (2010) MSM access to VCT in a conservative environment, case of Egypt. Abstract no WEPE0318 AIDS 2010 - XVIII International AIDS Conference. Vienna, Austria.

7. Egypt Ministry of Health and Population National AIDS Program (2006) HIV/AIDS biological and behavioral surveillance survey, Round I, Summary report Egypt 2006. Cairo, Egypt.

8. Shalaby K, Lengsfeld T, Abolmagd S, Ramy H, Abdel-Rahman I, et al. (2010) A unique approach of comprehensive care for most-at-risk populations in conservative societies: the case of Egypt. Abstract no MOPE0308 AIDS 2010 - XVIII International AIDS Conference. Vienna, Austria

9. Soliman C, Rahman IA, Shawky S, Bahaa T, Elkamhawi S, et al. (2010) HIV prevalence and risk behaviors of male injection drug users in Cairo, Egypt. AIDS 24 Suppl 2: S33-38.

10. Abu-Raddad L, Akala FA, Semini I, Riedner G, Wilson D, et al. (2010) Characterizing the HIV/AIDS epidemic in the Middle East and North Africa: Time for Strategic Action. Middle East and North Africa HIV/AIDS Epidemiology Synthesis Project. World Bank/UNAIDS/WHO Publication. Washington DC: The World Bank Press.

11. Farhoudi B, Montevalian A, Motamedi M, Khameneh MM, Mohraz M, et al. (2003) Human immunodeficiency virus and HIV - associated tuberculosis infection and their risk factors in injecting drug users in prison in Iran. Iran Ministry of Health, Tehran, Iran.

12. Kheirandish P, Seyedalinaghi SA, Hosseini M, Jahani MR, Shirzad H, et al. (2010) Prevalence and correlates of HIV infection among male injection drug users in detention in Tehran, Iran. J Acquir Immune Defic Syndr 53: 273-275.

13. Iran Ministry of Health (2006) Treatment and medical education. Islamic Republic of Iran HIV/AIDS situation and response analysis. Tehran, Iran.

14. Glareh Mostashari, Mojgan Darabi (2006) Summary of the Iranian Situation on HIV Epidemic. NSP Situation Analysis. UNODC, Tehran, Iran.

15. S. Momtazi, M. Fallahnejad, A. Shoghli, N. Musavinasab, Tavassoli S (2010) HIV high risk behavior in a sample of Iranian injection drug users. Abstract no. TUPE0338. AIDS 2010 - XVIII International AIDS Conference. Vienna, Austria.

16. Zamani S, Kihara M, Gouya MM, Vazirian M, Nassirimanesh B, et al. (2006) High prevalence of HIV infection associated with incarceration among community-based injecting drug users in Tehran, Iran. J Acquir Immune Defic Syndr 42: 342-346.

17. Zamani S, Radfar R, Nematollahi P, Fadaie R, Meshkati M, et al. (2010) Prevalence of HIV/HCV/HBV infections and drug-related risk behaviours amongst IDUs recruited through peer-driven sampling in Iran. Int J Drug Policy 21: 493-500.

18. Narenjiha H, Rafiey H, Baghestani A, et al. (2005) [Rapid Situation Assessment of Drug Abuse and Drug Dependence in Iran, DARIUS Institute - Draft Report] (Persian). Tehran, Iran.

19. Mirahmadizadeh AR, Majdzadeh R, Mohammad K, MH F (2009) Prevalence of HIV and Hepatitis C Virus Infections and Related Behavioral Determinants among Injecting Drug Users of Drop-in Centers in Iran. Iranian Red Crescent Medical Journal 11: 325-329.

20. Razzaghi E, Rahimi A, Hosseini M, A. C (1999) Rapid Situation Assessment (RSA) of Drug Abuse in Iran. Tehran: Prevention Department, State Welfare Organization, Ministry of Health, I.R. of Iran and United Nations International Drug Control Program.

21. Mahfoud Z, Afifi R, Ramia S, El Khoury D, Kassak K, et al. (2010) HIV/AIDS among female sex workers, injecting drug users and men who have sex with men in Lebanon: results of the first biobehavioral surveys. AIDS 24 Suppl 2: S45-54.

22. Aaraj E Report on the situation analysis on vulnerable groups in Beirut, Lebanon. Lebanon Ministry of Health, Beirut, Lebanon.

23. Ministere de la Sante au Maroc, Direction de l’Épidémiologie et de Lutte contre les Maladies, Programme de lutte contre la toxicomanie (2005) Evaluation rapide de la situation sur le risque d’infection à VIH en relation avec l’usage des drogues injectées et injectables et à problème au Maroc (French) [Rapid situation assessment on the risk of HIV infection associated with the use of injected, injectable, and other drugs in Morocco]. Rabat, Morocco.

24. Oman Ministry of Health (2006) HIV Risk among Heroin and Injecting Drug Users in Muscat, Oman. Quantitative Survey. Preliminary Data. Muscat, Oman.

25. Bokhari A, Nizamani NM, Jackson DJ, Rehan NE, Rahman M, et al. (2007) HIV risk in Karachi and Lahore, Pakistan: an emerging epidemic in injecting and commercial sex networks. Int J STD AIDS 18: 486-492.

26. Pakistan National AIDS Control Program (2005) Integrated Biological & Behavioral Surveillance 2004-05. Report of the Pilot Study in Karachi & Rawalpindi. Ministry of Health Canada-Pakistan HIV/AIDS Surveillance Project.

27. Hawkes S, Collumbien M, Platt L, Lalji N, Rizvi N, et al. (2009) HIV and other sexually transmitted infections among men, transgenders and women selling sex in two cities in Pakistan: a cross-sectional prevalence survey. Sex Transm Infect 85 Suppl 2: ii8-16.

28. Pakistan National AIDS Control Program (2008) HIV Second Generation Surveillance In Pakistan. National Report Round III. Canada-Pakistan HIV/AIDS Surveillance Project. National Aids Control Program, Ministry Of Health, Pakistan.

29. Pakistan National AIDS Control Program (2005) HIV Second Generation Surveillance In Pakistan. National Report Round I. Canada-Pakistan HIV/AIDS Surveillance Project. National Aids Control Program, Ministry Of Health, Pakistan.

30. Pakistan National AIDS Control Program (2006-07) HIV Second Generation Surveillance In Pakistan. National Report Round II. Canada-Pakistan HIV/AIDS Surveillance Project. National Aids Control Program, Ministry Of Health, Pakistan.

31. Parviz S, Fatmi Z, Altaf A, McCormick JB, Fischer-Hoch S, et al. (2006) Background demographics and risk behaviors of injecting drug users in Karachi, Pakistan. Int J Infect Dis 10: 364-371.

32. Haque N, Zafar T, Brahmbhatt H, Imam G, ul Hassan S, et al. (2004) High-risk sexual behaviours among drug users in Pakistan: implications for prevention of STDs and HIV/AIDS. Int J STD AIDS 15: 601-607.

33. Emmanuel F, Akhtar S, Attarad A, Kamran C (2004) HIV risk behavior and practices among heroin addicts in Lahore, Pakistan. Southeast Asian J Trop Med Public Health 35: 940-948.

34. Emmanuel F, Fatima M (2008) Coverage to curb the emerging HIV epidemic among injecting drug users in Pakistan: delivering prevention services where most needed. Int J Drug Policy 19 Suppl 1: S59-64.

35. Kuo I, ul-Hasan S, Galai N, Thomas DL, Zafar T, et al. (2006) High HCV seroprevalence and HIV drug use risk behaviors among injection drug users in Pakistan. Harm Reduct J 3: 26.

36. Ministere de la Sante Publique en Tunisie, Association Tunisienne de Lutte Contre les MST et le SIDA (2010) Enquête sérocomportementale auprès des hommes ayant des rapports sexuels avec des hommes en Tunisie (French) [Biobehavioral survey among men who have sex with men in Tunisia]. Tunis, Tunisia.

37. Hsairi M, Ben Abdallah S (2007) Analyse de la situation de vulnérabilité vis-à-vis de l’infection à VIH des hommes ayant des relations sexuelles avec des hommes. Rapport Final, version abrégée (French) [Analysis of the HIV vulnerability settings of men who have sex with men. Final report, short version]. Tunisia Ministry of Health, Tunis, Tunisia.

38. Syria Mental Health Directorate, Syria National AIDS Programme (2008) Assessment of HIV Risk and Sero-prevalence among Drug Users in Greater Damascus. Syrian Ministry of Health. UNODC. UNAIDS. Damascus, Syria
