# Supplementary material for: Are HIV Epidemics among Men Who Have Sex with Men Emerging in the Middle East and North Africa?: A Systematic Review and Data Synthesis
Source: PLoS Med. 2011 Aug 2;8(8):e1000444. doi: 10.1371/journal.pmed.1000444 (PMC3149074; doi:10.1371/journal.pmed.1000444)
Supplement: Table S6 — Description of various sampling methodologies used in surveying MSM in MENA. (0.04 MB DOC) [file pmed.1000444.s006.doc]

**Table S6.** Description of various sampling methodologies used in surveying MSM in MENA.

| **Sampling techniques** | **Description** | **Probability-based** |
| --- | --- | --- |
| Convenience sampling (CvS) | Subjects are selected because of their convenient accessibility and proximity to the researcher. One example is facility-based sampling such as sampling MSM from STI clinics or through NGOs and VCT facilities. | No |
| Snow-ball sampling (SBS) | An initial group of subjects, ‘seeds’, are *conveniently* identified. These will in turn help recruit future subjects engaging in the same types of behavior from among their acquaintances and networks. | No |
| One-stage cluster sampling (CS) | A number of social settings or other sub-groupings are randomly selected from a population, following which all the subjects within the selected sub-groupings are recruited. CS has been applied to HSWs in Pakistan where a random sample of *gurus* (mentor/pimp) is selected from a list identified through ethnographic mapping, and then all HSWs under the selected *gurus* are included in the sample. | Yes |
| Respondent-driven sampling (RDS) | RDS is a recent sophisticated variant of SBS and considered state of the arts in terms of sampling MSM. It is a chain-referral strategy relying on peer-recruitment; but the recruitment process has strict conditions and follows a systematic protocol using limited number of allowed recruitments per ‘seed’, which allows for the calculation of selection probabilities. | Yes |

This table describes the various sampling methodologies that were used to survey MSM in MENA in the studies using well-defined methodologies summarized in Table 3 of the main text. Table S6 also indicates which of these techniques are probability-based.
