# Supplementary material for: Are HIV Epidemics among Men Who Have Sex with Men Emerging in the Middle East and North Africa?: A Systematic Review and Data Synthesis
Source: PLoS Med. 2011 Aug 2;8(8):e1000444. doi: 10.1371/journal.pmed.1000444 (PMC3149074; doi:10.1371/journal.pmed.1000444)
Supplement: Text S2 — Details of data sources and search criteria. (0.06 MB DOC) [file pmed.1000444.s008.doc]

**Text S2: details on the search criteria**

The following material describes the different data sources and the details of the search criteria, for both the comprehensive review of HIV in MENA undertaken as part of the MENA HIV/AIDS Epidemiology Synthesis Project [1], and the specific men who have sex with men (MSM) in MENA search undertaken specifically for the present manuscript:

1. Comprehensive review of HIV data in MENA:

The following data sources were identified as part of our extensive search of relevant studies and databases in the MENA HIV/AIDS Epidemiology Synthesis Project:

1. Scientific literature search of PubMed (Medline) using a strategy with both free text and MeSH headings, and with no language or year limitations. The cut-off date of all the searches was April 19, 2011. Although most of the results of the searches have not been described in this article, the following set of criteria were used as part of the comprehensive search of the PubMed database that was conducted:
   - Studies of HIV infectious spread in its different modes of transmission under the strategy of ("HIV Seropositivity" OR "HIV" OR "HIV Infections") AND ("Middle East" OR "Islam" OR "Arabs" OR "Arab World" OR "Africa, Northern" OR "Mauritania" OR "Sudan" OR "Somalia" OR "Djibouti" OR "Pakistan"). This search yielded 1,194 publications.
   - Studies of sexual behavior and levels of risk behavior under the strategy of ("Sexual Behavior" OR "Sexual Partners" OR "Sexual Abstinence" OR "Unsafe Sex" OR "Sexology" OR "Reproductive Behavior" OR "Safe Sex" OR "Condoms" OR "Sex") AND ("Middle East" OR "Islam" OR "Arabs" OR "Arab World" OR "Africa, Northern" OR "Mauritania" OR "Sudan" OR "Somalia" OR "Djibouti" OR "Pakistan"). This search yielded 1,555 publications.
   - Studies of herpes simplex virus type 2 sero-prevalence under the strategy of ("Herpesvirus 2, Human" OR "Herpes Genitalis") AND ("Middle East" OR "Islam" OR "Arabs" OR "Arab World" OR "Africa, Northern" OR "Mauritania" OR "Sudan" OR "Somalia" OR "Djibouti" OR "Pakistan"). This search yielded 36 publications.
   - Studies of human papillomavirus and cervical cancer under the strategy of "Papillomavirus Infections" OR " Uterine Cervical Neoplasms") AND ("Middle East" OR "Islam" OR "Arabs" OR "Arab World" OR "Africa, Northern" OR "Mauritania" OR "Sudan" OR "Somalia" OR "Djibouti" OR "Pakistan"). This search yielded 337 publications.
   - Studies of bacterial sexually transmitted infections under the strategy of ("Chlamydia" OR "Chlamydia Infections" OR "Chlamydia trachomatis" OR "Gonorrhea" OR "Neisseria gonorrhoeae" OR "Syphilis" OR "Vaginosis, Bacterial" OR "Pelvic Inflammatory Disease") AND ("Middle East" OR "Arabs" OR "Islam" OR "Arab World" OR "Africa, Northern" OR "Mauritania" OR "Sudan" OR "Somalia" OR "Djibouti" OR "Pakistan"). This search yielded 574 publications.
   - Studies of hepatitis C virus under the strategy of ("Hepatitis C" OR "Hepatitis C Antibodies" OR "Hepatitis C Antigens") AND ("Middle East" OR "Islam" OR "Iran" OR "Arabs" OR "Arab World" OR "Africa, Northern" OR "Mauritania" OR "Sudan" OR "Somalia" OR "Djibouti" OR "Pakistan"). This search yielded 966 publications.
2. Peer-reviewed publications published in local and regional research journals not indexed in PubMed, but identified most often through Google Scholar.
3. Country-level reports and databases, governmental and non-governmental organizations’ studies and publications, as well as other institutional reports related to HIV and STIs in MENA. These include also countries’ case notification reports.
4. International organizations’ reports and databases related to HIV and other STIs. Among the organizations from which we obtained reports and data are UNAIDS, WHO, World Bank, United Nations Children's Fund (UNICEF), United Nations Office on Drugs and Crime (UNODC), International Agency for Research on Cancer (IARC), International Organization for Migration (IOM), International Centre for Prison Studies (ICPS), Office of the UN High Commissioner for Refugees (UNHCR), Population Reference Bureau (PRB) and Family Health International (FHI). The US Census Bureau database of HIV/AIDS, a compilation of global HIV prevalence studies irrespective of collection methodology, was searched [2], so was the WHO/EMRO HIV testing database [3]. Demographic and Health Survey (DHS) reports of MENA countries were also reviewed.
5. Consultations with key experts, public health officials, researchers, and academics in the region and beyond.
6. Specific data about MSM in MENA:

The above generic search was complemented by a specific search of all data on MSM in MENA in the biomedical literature as of April 19, 2011 using the following data sources:

1. The Pubmed database using MeSH headings and with no language or year limitations. The following set of criteria were used in the search:
   - Studies of male same-sex sexual behavior in MENA under the strategy of (("Homosexuality" OR "Bisexuality") AND ("Middle East" OR "Islam" OR "Arabs" OR "Arab World" OR "Africa, Northern" OR "Mauritania" OR "Sudan" OR "Somalia" OR "Djibouti" OR "Pakistan"). This search yielded 118 publications.
   - Studies of HIV among MSM in MENA under the strategy of (("HIV" OR "HIV Seropositivity" OR "HIV Infections") AND ("Homosexuality" OR "Bisexuality") AND ("Middle East" OR "Islam" OR "Arabs" OR "Arab World" OR "Africa, Northern" OR "Mauritania" OR "Sudan" OR "Somalia" OR "Djibouti" OR "Pakistan"). This search yielded 46 publications.

The criteria ("Homosexuality" OR "Bisexuality") was also searched in combination with each individual country’s name for more sensitivity, although these specific searches did not retrieve any new articles that were not identified in the above two searches using the broader MeSH terms defining the geographical boundaries of MENA. Furthermore, all of the studies identified in the *HIV among MSM in MENA* search, and almost all relevant studies that were retrieved from the *male same-sex sexual behavior in MENA* search, were already covered by the wide-umbrella search in part A above. However, these specific searches were undertaken and respective citations reviewed for further reassurance that no relevant MSM data in MENA were missed.

1. The Embase database using an *explosion advance search* with the following criteria and *emtree* terms: *('homosexuality' OR 'bisexuality') AND ('afghanistan' OR 'algeria' OR 'bahrain' OR 'djibouti' OR 'egypt' OR 'iran' OR 'iraq' OR 'jordan' OR 'kuwait' OR 'lebanon' OR 'libya' OR 'morocco' OR 'oman' OR 'pakistan' OR 'qatar’ OR 'saudi arabia' OR 'somalia' OR 'sudan' OR 'syria' OR 'tunisia' OR 'united arab emirates' OR 'palestine' OR 'yemen').* This search yielded 85 publications.
2. The WHO African Index Medicus (AIM) [4] using the following criteria and keywords:
   - (‘homosexuality’ OR ‘bisexuality’) in combination with each of the African countries in MENA, namely Algeria, Djibouti, Egypt, Libya, Morocco, Somalia, Sudan, and Tunisia. This search yielded zero publications.
   - ‘HIV’ in combination with each of the above African countries in MENA. This search was undertaken to ensure no relevant studies were missed as the ‘homosexuality or bisexuality’ search above did not yield any records and there were concerns as to the specificity of the search engine of the database. This search yielded 7 publications.
3. The WHO Index Medicus for the Eastern Mediterranean Region (IMEMR) [5] using the following keywords: ‘homosexuality’, ‘homosexual’, ‘bisexuality’, and ‘bisexual’. These searches yielded 20, 7, 0, and 7 publications respectively.
4. The Scientific and Technical Egyptian Bibliographic Database (STEB) [6] using the following criteria and keywords:
   - (‘homosexuality’ OR ‘homosexual). This search yielded 25 publications.
   - ‘HIV’ in combination with each of the 23 MENA countries listed in Text S3. This search yielded 12 publications and was undertaken to complement the above search as there were concerns as to the specificity of the search engine of the database.

**References**
