# Supplementary material for: Are HIV Epidemics among Men Who Have Sex with Men Emerging in the Middle East and North Africa?: A Systematic Review and Data Synthesis
Source: PLoS Med. 2011 Aug 2;8(8):e1000444. doi: 10.1371/journal.pmed.1000444 (PMC3149074; doi:10.1371/journal.pmed.1000444)
Supplement: Text S3 — List of MENA countries. (0.04 MB DOC) [file pmed.1000444.s009.doc]

**Text S3: List of MENA Countries**

The study covered all 23 countries included in the MENA definitions of the three partner organizations; the World Bank, UNAIDS, and WHO/EMRO. These countries include:

- Afghanistan
- Algeria
- Bahrain
- Djibouti
- Egypt
- Iran
- Iraq
- Jordan
- Kuwait
- Lebanon
- Libya
- Morocco
- Oman
- Pakistan
- Qatar
- Saudi Arabia
- Somalia
- Sudan
- Syria
- Tunisia
- United Arab Emirates
- West Bank and Gaza (Occupied Palestinian Territories)
- Yemen

Considering geographic proximity and similarity in the socio-cultural context, data were occasionally included on Mauritania, native Palestinian population in Israel, and Turkey.
